# Nationwide spatiotemporal drug resistance genetic profiling from over three decades in Indian *Plasmodium falciparum* and *Plasmodium vivax* isolates

**DOI:** 10.1186/s12936-023-04651-x

**Published:** 2023-08-15

**Authors:** Loick P. Kojom Foko, Geetika Narang, Jahnvi Jakhan, Suman Tamang, Amit Moun, Vineeta Singh

**Affiliations:** Parasite & Host Biology Group, ICMR-National Institute of Malaria Research, Dwarka, Sector 8, New Delhi, 110077 India

**Keywords:** Malaria, *P. falciparum*, *P. vivax*, Drug resistance, Molecular markers, India

## Abstract

**Background:**

Drug resistance is a serious impediment to efficient control and elimination of malaria in endemic areas.

**Methods:**

This study aimed at analysing the genetic profile of molecular drug resistance in *Plasmodium falciparum* and *Plasmodium vivax* parasites from India over a ~ 30-year period (1993–2019). Blood samples of *P. falciparum* and/or *P. vivax*-infected patients were collected from 14 regions across India. Plasmodial genome was extracted and used for PCR amplification and sequencing of drug resistance genes in *P. falciparum* (*crt*, *dhps*, *dhfr*, *mdr1*, *k13*) and *P. vivax* (*crt-o*, *dhps*, *dhfr*, *mdr1*, *k12*) field isolates.

**Results:**

The double mutant *pfcrt* **S**VMN**T** was highly predominant across the country over three decades, with restricted presence of triple mutant CV**IET** from Maharashtra in 2012. High rates of *pfdhfr*-*pfdhps* quadruple mutants were observed with marginal presence of “fully resistant” quintuple mutant AC**IRN**I-IS**GE**AA. Also, resistant *pfdhfr* and *pfdhps* haplotype has significantly increased in Delhi between 1994 and 2010. For *pfmdr1*, only 86Y and 184F mutations were present while no *pfk13* mutations associated with artemisinin resistance were observed. Regarding *P. vivax* isolates, the *pvcrt-o* K10 “AAG” insertion was absent in all samples collected from Delhi in 2017. *Pvdhps* double mutant S**GN**AV was found only in Goa samples of year 2008 for the first time. The *pvmdr1* 908L, 958M and 1076L mutations were highly prevalent in Delhi and Haryana between 2015 and 2019 at complete fixation. One nonsynonymous novel *pvk12* polymorphism was identified (K264R) in Goa.

**Conclusions:**

These findings support continuous surveillance and characterization of *P. falciparum* and *P. vivax* populations as proxy for effectiveness of anti-malarial drugs in India, especially for independent emergence of artemisinin drug resistance as recently seen in Africa.

**Supplementary Information:**

The online version contains supplementary material available at 10.1186/s12936-023-04651-x.

## Background

The control of malaria still continues to pose a problem in endemic countries, especially due to emergence and spread of drug resistant *Plasmodium* parasites across the globe [1]. *Plasmodium falciparum* and *Plasmodium vivax* are responsible for the bulk of global morbidity and mortality statistics [1, 2]. The most dangerous human malaria species is *P. falciparum* with high risk of severe complications if not treated promptly [3]. *Plasmodium vivax* is the most geographically distributed species, mainly encountered in South East Asia (SEA), Latin Americas, and the horn of Africa. India accounted for ~ 79% of cases and ~ 83% of deaths reported in SEA in 2021 [1, 4]. Recently, there are increasing reports on the ability of *P. vivax* parasites to induce severe malaria and deaths in some patients [5–7].

**Fig. 1:**
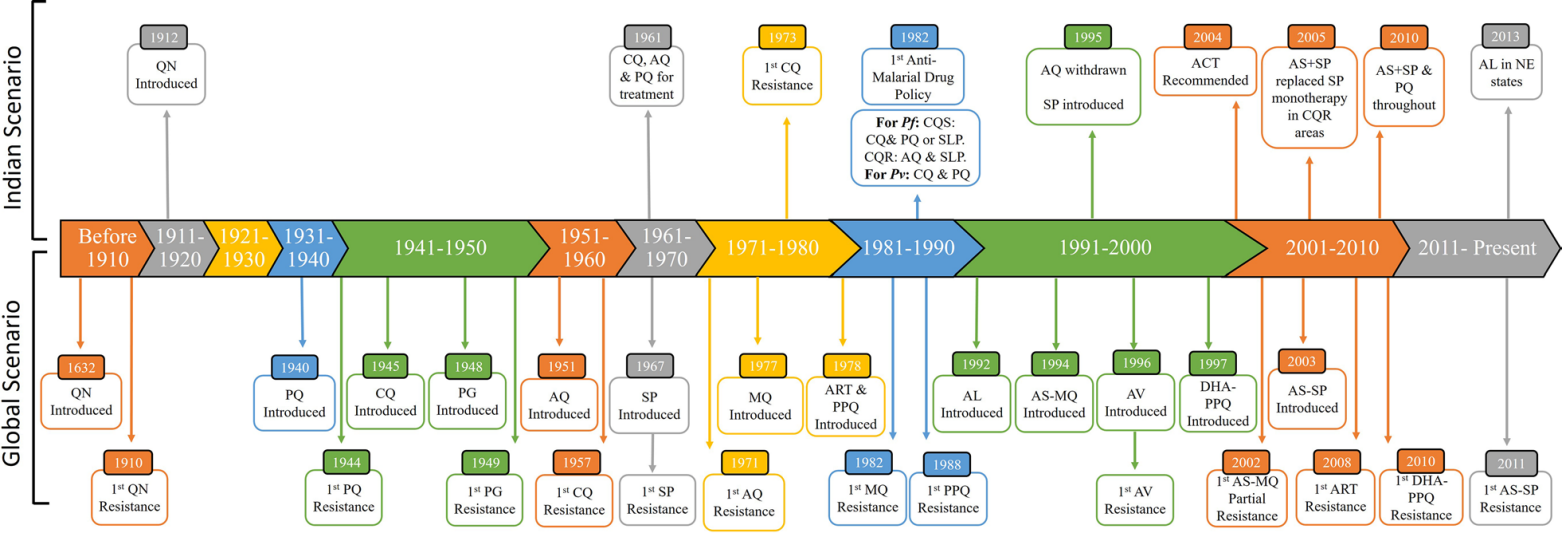
Timeline of introduction and appearance of resistance to main anti-malarial drugs in the world and India. *QN* quinine, *PPQ* piperaquine, *PQ* primaquine, *CQ* chloroquine, *PG* proguanil, *MQ* mefloquine, *ART* artemisinin, *SP* sulfadoxine–pyrimethamine, *AQ* amodiaquine, *ACT* artemisinin based combination therapy, *AS* artesunate, *AL* artemether–lumefantrine, *SLP* sulfalene–pyrimethamine, *CQR* chloroquine-resistant, *NE* North east states (Sources: [15, 25, 27–32])

*Plasmodium falciparum* parasites have developed resistance phenotypes to all major anti-malarial drugs, such as chloroquine (CQ), mefloquine (MQ), sulfamides, artemisinin (ART) and its derivatives (Fig. 1) [8, 9]. In contrast, some studies evidenced the emergence of *P. vivax* parasites resistant to fewer drugs, such as CQ and MQ [10, 11]. The current first-line uncomplicated *P. falciparum* malaria treatment policies rely on artemisinin-based combination therapy (ACT) which consist of a combination drugs (i.e., ART derivatives) with slow-acting drug referred to as partner drug [12]. Currently, six artemisinin-based combinations are recommended by the World Health Organization (WHO) for treating uncomplicated *P. falciparum* malaria viz. artesunate + sulfadoxine–pyrimethamine (AS + SP), artesunate + amodiaquine (AS + AQ), artesunate + pyronaridine (AS + PY), artemether + lumefantrine (AL), dihydroartemisinin + piperaquine (DHA + PPQ), and artesunate + mefloquine (AS + MQ) [1, 8]. CQ is commonly used for treating clinical *P. vivax* infections. The WHO recommended to associate CQ with primaquine (PQ), a hypnozoiticidal drug, given the ability of *P. vivax* parasites to induce relapses due to reactivation of dormant liver stages (i.e., hypnozoites). The association CQ + PQ, also known as radical cure, guarantees treatment of current infection and prevent recurrent infection due to relapses by killing blood and liver parasite stages [13, 14].

The emergence and spread of ACT-resistant *P. falciparum* parasites in SEA has profoundly thwarted malaria control and elimination objectives in the region [15, 16]. More recently, independent appearance of ACT-resistant *P. falciparum* parasites from two African countries Rwanda and Uganda [17–19]; has given basis to fear the spread of ACT-resistance in Africa in future. In this context, anti-malarial drug resistance surveillance is a key component to successful malaria control and elimination. Several tools have been developed for *Plasmodium* drug resistance and these consist of (i) in vivo drug efficacy studies, (ii) in vitro assessment of drug susceptibility in parasites, and (iii) tracking of molecular markers associated with drug resistance [20]. The latter tools are largely used for drug resistance surveillance given the high cost of in vivo studies and lack of standardization of in vitro studies [20]. Also, molecular tools are much easier to implement and/or scale-up, and give prevalence estimates of drug resistant *Plasmodium* parasites over time and space with possibility for within and between study comparisons [20].

In India, *P. falciparum* and *P. vivax* are the two predominant *Plasmodium* species with prevalence ratio varying between states, but overall ratio close to one [21–23]. Clinical spectrum of malaria infections with *P. falciparum* and *P. vivax* ranges from asymptomatic to severe malaria [3, 5]. India has continuously modified and adapted national drug policies since 1982 to efficiently and timely control anti-malarial drug resistance (Fig. 1). The current treatment policy endorses treatment of (i) uncomplicated *P. falciparum* malaria with either artesunate + lumefantrine (AL) in North Eastern states or artesunate + sulfadoxine + pyrimethamine (AS + SP) in rest of states, accompanied by single dose PQ, (ii) uncomplicated *P. vivax* malaria with CQ + PQ, and (iii) severe malaria with quinine, artemether, artesunate, or artemether for 48 h, followed by quinine or above mentioned a state-specific artemisinin-based combination [24]. In contrast, data on anti-malarial drug resistance in *P. falciparum* and *P. vivax* parasites are still insufficient and fragmented in India [8, 25, 26]. The present study describes the spatial and temporal distribution of main putative molecular markers associated with drug resistance in *P. falciparum* and *P. vivax* isolates collected between 1993 and 2019 among malarious patients from different areas of India.

## Methods

### Sample and diagnosis

This study has been approved by the institutional review board of ICMR-National Institute of Malaria Research (NIMR), India. Malaria samples were collected between 1993 and 2019 from different field sites and health facilities (hospitals and primary health centres) of different states and union territories (Additional file 1). On field sites, malaria infection was first detected using rapid diagnostic tests (RDT), and confirmed microscopically and by PCR assay. Two RDTs namely SD Bioline *Pf*/*Pv*® (Standard Diagnostics, Inc., South Korea) and FalciVax™ (*Pf*/*Pv*) (Zephyr Biomedical, Verna, Goa, India) were used in this study. SD Bioline *Pf*/*Pv*® is a *P. falciparum* and *P. vivax* detecting RDT which targets *Pf*LDH + *Pv*LDH antigens. It has a panel detection score of 99.0% and 97.1% at 200 parasites/µL for *P. falciparum* and *P. vivax,* respectively. FalciVax™ (*Pf*/*Pv*) is a *P. falciparum* and *Pv*/*Pvom* detecting RDT which targets *Pf*HRP2 + *Pv*LDH antigens, with a panel detection score of 95.0% and 100% at 200 parasites/µL for *P. falciparum* and *P. vivax,* respectively [33].

**Fig. 2:**
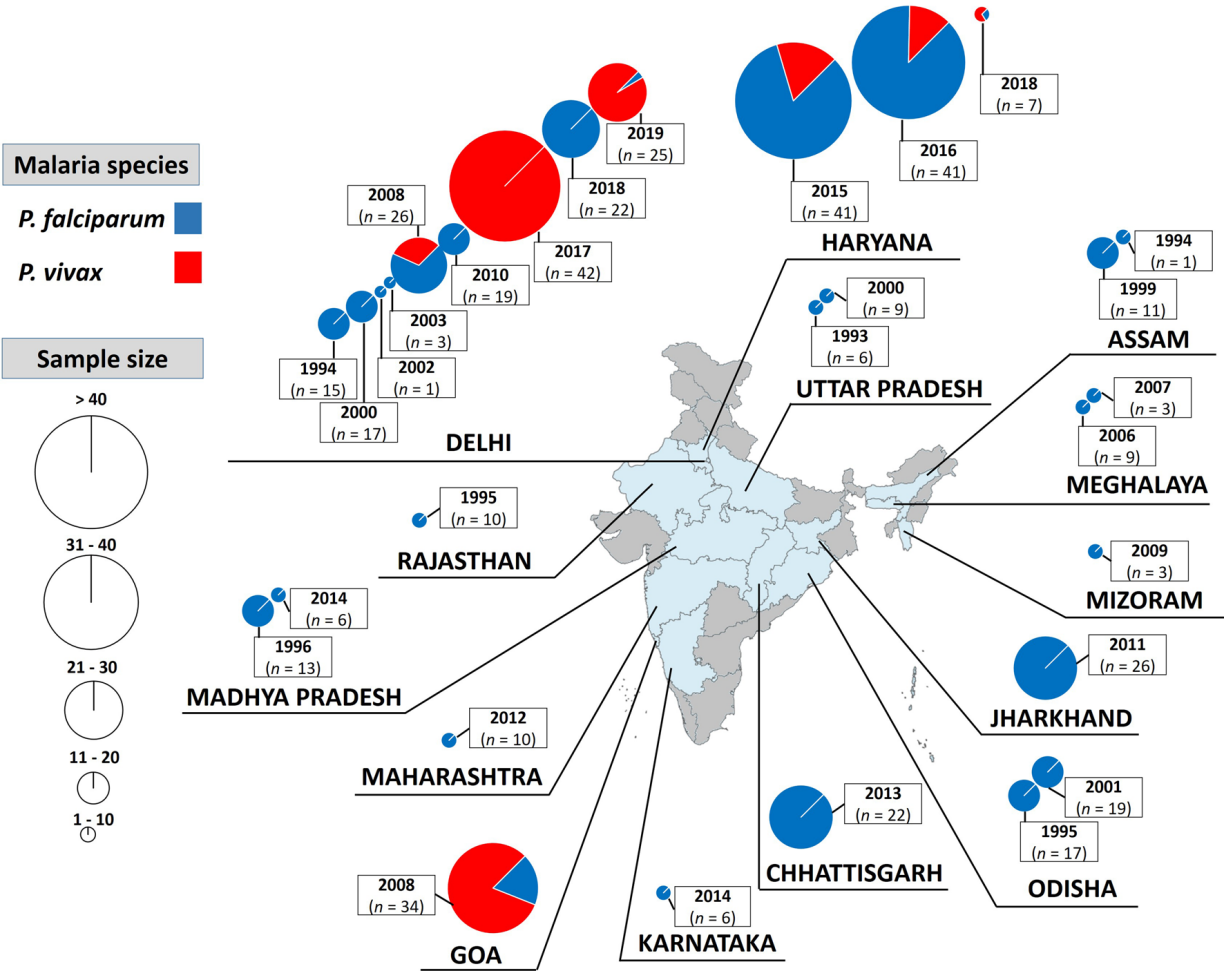
Map of India showing study areas where *Plasmodium* isolates were collected. Each pie chart represents the total number of isolates analysed. *P. falciparum* (blue) and *P. vivax* (red). The size of pie chart is proportional to sample size. The map depicted here is taken from official website of Ministry of External Affairs, Government of India (https://mea.gov.in/india-at-glance.htm, accessed 15/11/2021)

The study samples comprised of both; clinically asymptomatic and symptomatic individuals of all age living in rural, semi-urban and urban areas of India. A total of 16 areas belonging to 14 states and union territories were study sites in the present study (Fig. 2). Details on urbanization level and malaria endemicity of study areas are presented in Additional file 2. The plasmodial DNA was extracted using QIAamp® DNA Mini Kit (Qiagen, Valencia, California, USA) as per manufacturer’s instructions in a final elution volume of 70 µL buffer (10 mM Tris–HCl; 0.5 mM EDTA; pH 9.0), and then stored at − 20 °C until needed. Plasmodial speciation was performed using polymerase chain reaction (PCR) protocols targeting the 18S subunit ribosomal unit gene of *P. falciparum* and *P. vivax* (Table 1) [34]. DNA sample was mixed in 25 µL PCR reaction containing 12.5 µL of 2X Go Taq green master mix (Promega Corporation, USA), 1 µL of each primer (10 µM), 1–2 µL DNA template, and free-nuclease water Q.S. PCR amplicons were loaded on 2% agarose gel pre-stained with ethidium bromide at 72 V for 1 h, and then visualized using an ultraviolet trans-illuminator. *P. falciparum* and *P. vivax* infections were confirmed by the presence of PCR bands of 205 bp and 120 bp, respectively [34].

**Table 1:** Main mutations in *Pf* and *Pv* drug resistance genes analyzed in the study

Genes	Chromosome	Validated/candidate or putative markers	Main mutations investigated	Antimalarial drugs/classes
*pfcrt*	7	Validated	72S, 73K, 74I, 75E, 76T	CQ, AQ
*pfdhfr*	4	Validated	16V, 50R, 51I, 59R, 108N, 164L	Pyrimethamine, cycloguanil
*pfdhps*	8	Validated	431V, 436A/F, 437G, 540E, 581G, 613S/T	Sulfonamide, sulfadoxine, sulfone, dapsone
*pfmdr1*	5	Validated	86Y, 124F, 1034C, 1042D, 1246Y	CQ, AQ, L, MEF
*pfk13*	13	Validated	F446I, N458Y, C469Y, M476I, Y493H, R539T, I543T, P553L, R561H, P574L, C580Y, R622I and A675V	ART and its derivatives
		Candidate	P441L, G449A, C469F, A481V, R515K, P527H, N537I/D, G538V and V568G	
*pvcrt-o*	1	Putative	K10 (AAG insertion)	–
*pvdhfr*	5	Putative	57L/I, 58R, 61M, 117T/N, 173F	–
*pvdhps*	14	Putative	382F/A/C, 383G, 399I, 512M, 525G, 553G, 555R, 585G, 661V	–
*pvmdr1*	10	Putative	845F, 861E, 898E, 908L, 958M, 976F/V, 1076L/I/T	–
*pvk12*	12	Putative	88S, 124I, 552I, 581R, 697S	–

ART: artemisinin; AQ: amodiaquine; CQ: chloroquine; L: lumefantrine; MEF: mefloquine; *Pf*: *P. falciparum*; *Pv*: *P. vivax*; *crt*: chloroquine resistant transporter gene; *crt-o*: chloroquine resistant transporter orthologue gene; *dhfr*: dihydrofolate reductase gene; *dhps*: dihydropteroate synthase gene; *mdr1*: multidrug resistance protein 1 gene; *k12*: Kelch12 gene; *k13*: Kelch13 gene

### Amplification of *P. falciparum* and *P. vivax* drug resistance genes

Single-step and nested PCR protocols were used to amplify five genes associated with drug resistance in *P. falciparum* parasites viz. chloroquine resistance transporter (*pfcrt*), dihydrofolate reductase (*pfdhfr*), dihydropteroate synthase (*pfhdps*), multidrug resistance protein 1 (*pfmdr1*), and Kelch protein (*pfk13*) [8]. Also, orthologues of these genes in *P. vivax* isolates were analysed viz *pvcrt-o*, *pvdhfr*, *pvdhps*, *pvmdr1* and *pvk12,* using published and developed singe-step and nested PCR protocols (Additional file 3).

### Sequencing and SNP analysis

The amplicons were purified using GeneJet purification kit (Thermofisher) and sequenced in both directions based on Sanger dideoxy method. Sequencing were performed on in-house ABI 3730XL DNA analyzer (Applied Biosystem) with BigDye Terminator v3.1 sequencing kit (Applied Biosystem). Nucleotide and deduced amino acid of gene sequences were aligned and compared with references by using CLUSTALW program of MEGA X [35].

*Plasmodium falciparum* and *P. vivax* sequences of drug resistance genes were analysed in comparison with those of reference strains. The reference *P. falciparum* strains accession numbers were PF3D7_0709000 for *pfcrt*, PF3D7_0417200 for *pfdhfr*, PF3D7_0810800 for *pfdhps*, PF3D7_0523000 for *pfmdr1,* and PF3D7_1343700 for *pfk13*. The reference *P. vivax* strains used were PVX_087980 for *Pvcrt-o*, PVX_089950 for *pvdhfr*, PVX_123230 for *pvdhps*, PVX_080100 for *pvmdr-1,* and PVX_083080 for *pvkelch12*. The phylogenetic relatedness of *P. falciparum* and *P. vivax* isolates was done through BLAST of drug resistance sequences. After sequence alignment nucleotide positions which displayed two peaks at one locus in chromatogram were noted as ‘‘mixed’’ and excluded from further analysis. Known point mutations in *P. falciparum* genes associated with anti-malarial drug resistance (CQ, SP, ART and its derivatives), and novel mutations were identified using their corresponding amino acids and haplotypes [8, 36]. Regarding *P. vivax* isolates, putative drug resistance-associated mutations were also investigated [37–40] (Table 1). The proportions of each validated mutation and putatively associated with anti-malarial drug resistance in *P. falciparum* and *P. vivax* isolates were calculated by areas and year of collection. Similar analysis was made for drug resistance genotypes and haplotypes.

### Statistical analysis

Data were keyed, coded and verified for consistency in an Excel spreadsheet (Microsoft Office, USA), and then exported to GraphPad v8.0.2 for Windows (GraphPad PRISM, Inc., San Diego, CA, USA) and StatView v5.0 for Windows (SAS Institute, Inc., Chicago, USA) for statistical analysis. Data were summarized as percentages in tables and graphics. Changes in the prevalence of alleles and haplotypes over time were evaluated for statistical significance using Pearson’s independence χ^2^ statistics for trend. Statistical significance was set at *p* < 0.05.

## Results

### *Plasmodium* samples

**Fig. 3:**
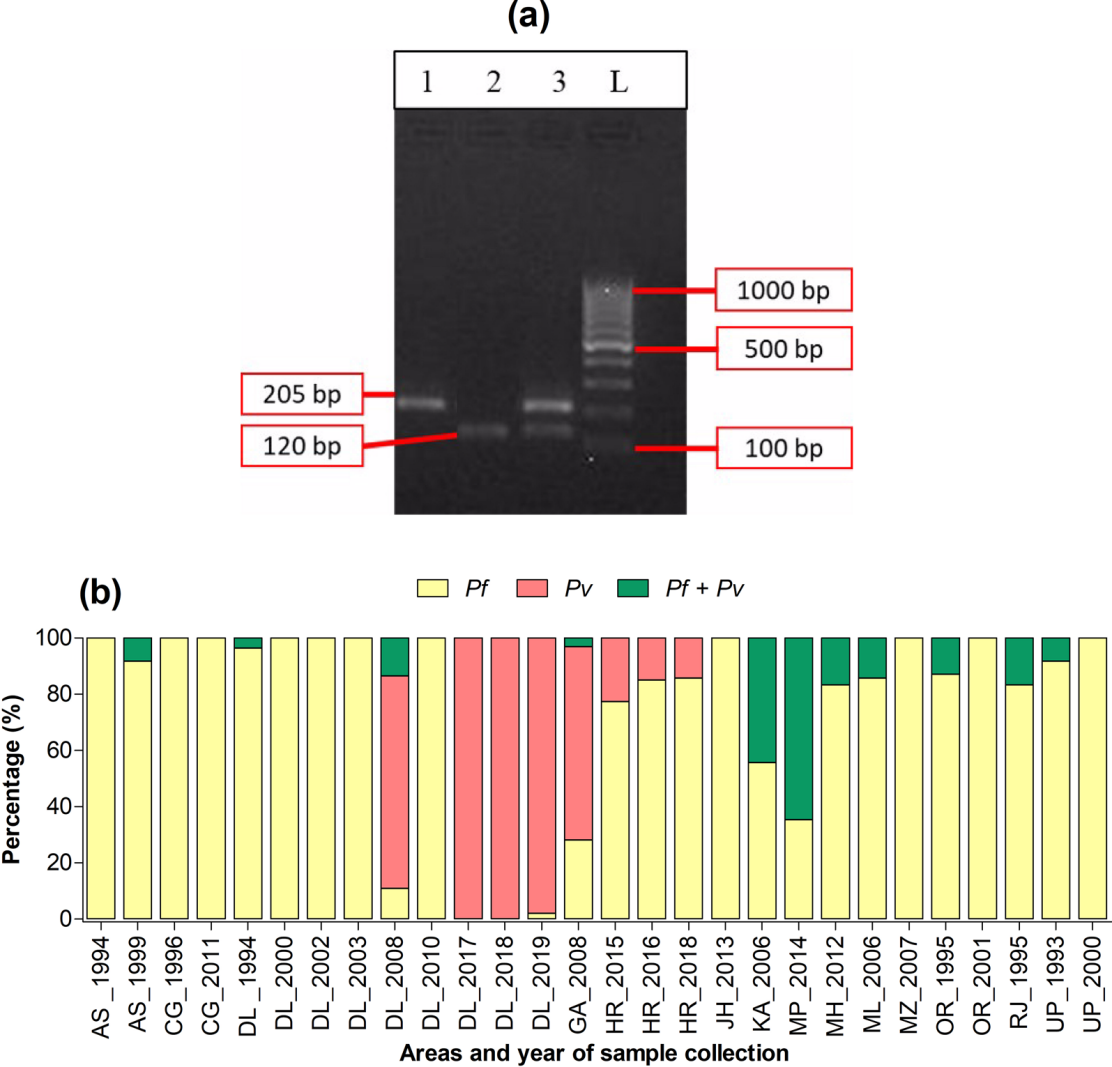
Electrophoresis gel depicting *P. falciparum* mono-infection, *P. vivax* mono-infection and mixed infection (**a**), and proportion of types of infections with *P. falciparum* and/or *P. vivax* (**b**). In **a**, the gel image is showing the 18S RNA PCR amplification of *Plasmodium* species. Lane 1: *Plasmodium falciparum* (205 bp). Lane 2: *Plasmodium vivax* (120 bp). Lane 3: mixed infection (Both *P. falciparum* and *P. vivax*). Lane L: 100 bp Ladder. In **b**, the international codes of areas were used. *AS* Assam, *CG* Chhattisgarh, *DL* Delhi, *GA* Goa, *HR* Haryana, *JH* Jharkhand, *KA* Karnataka, *MH* Maharashtra, *ML* Meghalaya, *MP* Madhya Pradesh, *MZ* Mizoram, *OR* Orissa, *RJ* Rajasthan, *UP* Uttar Pradesh

A total of 593 samples were positive for *18S* genes, and infections with *P. falciparum* and *P. vivax* were found as either mono-infection or mixed infection (Fig. 3a and Additional file 4). The highest proportion of mixed infections were seen in Karnataka (64.7%) and Madhya Pradesh (44.4%) (Fig. 3b). Only mono-infections with *P. falciparum* or *P. vivax* were included in the study. The final number of samples included for each drug resistance gene varied from 30 to 318 based exclusion criteria (positive PCRs, good PCR bands, successful purification, successful sequencing, good quality sequencing) (Additional file 4). Additional file 5 depicts gel electrophoresis of *P. falciparum* and *P. vivax* drug resistance gene PCR results.

### Profiling of *P. falciparum* anti-malarial drug resistance genes

#### *Pfcrt*

**Fig. 4:**
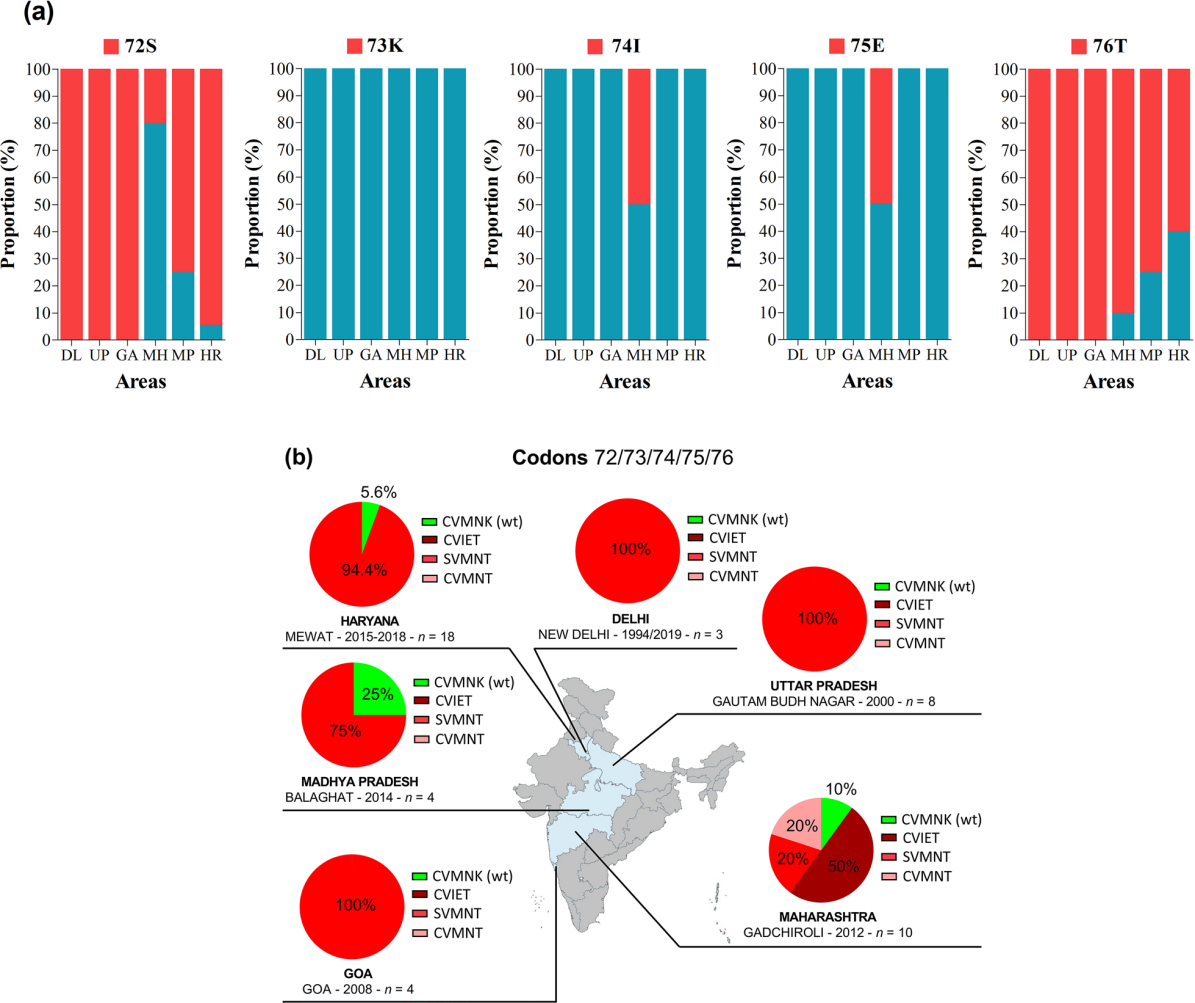
*Pfcrt* genotypes by year and area. **a** Proportion of 72S, 73K, 74I, 75E and 76T mutations in *pfcrt* gene, **b** proportion of *pfcrt* haplotypes. *Pf*: *P. falciparum*; *crt*: chloroquine resistant transporter gene. The international codes of areas were used. *DL* Delhi, *GA* Goa, *HR* Haryana, *MH* Maharashtra, *MP* Madhya Pradesh, *UP* Uttar Pradesh. In **a**, sample size was different for each area. DL (*n* = 3), GA (*n* = 4), HR (*n* = 18), MH (*n* = 10), MP (*n* = 4), UP (*n* = 8). In **b** the wild type is depicted in green while mutants are depicted in red and pink. The map depicted here is taken from official website of Ministry of External Affairs, Government of India (https://mea.gov.in/india-at-glance.htm, accessed 15/11/2021)

A total of 47 samples from Delhi, Haryana, Madhya Pradesh, Maharashtra, Goa, and Uttar Pradesh regions were analysed for the *pfcrt* gene. The analysis revealed high proportions of 72S and 76T mutations and absence of 73K mutation in samples from different parts of the country. All parasites from Delhi, Uttar Pradesh, and Goa were carrying these two mutations. The *pfcrt* 74I and 75E mutations were only reported from Maharashtra in equal proportion (50% each) (Fig. 4a). Three types of *pfcrt* genotypes viz. C_72_V_73_M_74_N_75_**T**_76_ (single mutant), **S**_72_V_73_M_74_N_75_**T**_76_ (double), and C_72_V_73_**I**_74_**E**_75_**T**_76_ (triple mutant) linked to anti-malarial resistance were reported (Fig. 3b). The double mutant **S**VMN**T** was predominant in most of the areas with proportions ranging from 75 to 100% between 1994 and 2019, with the exception of Maharashtra where 50% of isolates collected in 2012 had triple mutations (i.e., CV**IET**) (Fig. 4b). No novel mutation and synonymous mutations were found in *pfcrt* analysed sequences.

#### *Pfdhfr* and *pfdhps*

In total, 239 and 318 samples were successfully sequenced for *pfdhfr* and *pfdhps* genes (Additional file 5). Samples were collected from different regions of the country viz: Assam, Chhattisgarh, Delhi, Goa, Haryana, Jharkhand, Karnataka, Maharashtra, Meghalaya, Mizoram, Orissa, Rajasthan, and Uttar Pradesh.

**Fig. 5:**
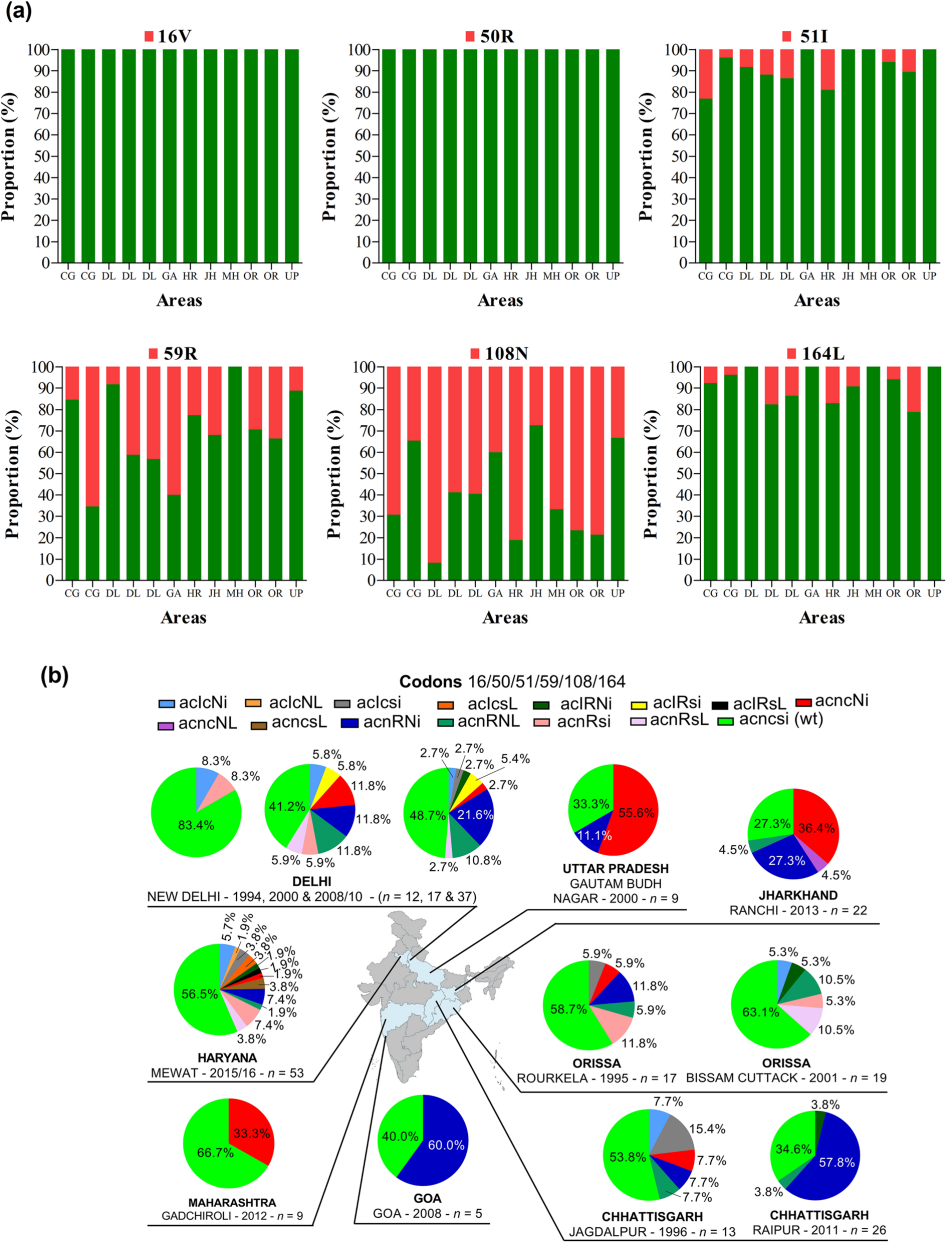
*Pfdhfr* genotypes by year and area. **a** Proportion of *pfdhfr* 16 V, 50R, 51I, 59R, 108N and 164L mutations, **b** proportion of *pfdhfr* haplotypes. *Pf*: *P. falciparum*; *dhfr*: dihydrofolate reductase gene; wt: wild type. The international codes of areas were used. *CG* Chhattisgarh, *DL* Delhi, *GA* Goa, *HR* Haryana, *JH* Jharkhand, *MH* Maharashtra, *MP* Madhya Pradesh, *OR* Orissa, *UP* Uttar Pradesh. In **a**, sample size was different for each area. CG (*n* = 13 and 26), DL (*n* = 12, 17, and 37), GA (*n* = 5), HR (*n* = 53), JH (*n* = 22), MH (*n* = 9), OR (*n* = 17 and 19), UP (*n* = 9). In **b** wild alleles are in lower case and mutant alleles are in upper case. The map depicted here is taken from official website of Ministry of External Affairs, Government of India (https://mea.gov.in/india-at-glance.htm, accessed 15/11/2021)

On analysis of the *pfdhfr* gene, four of the six validated SNPs (i.e., 51I, 59R, 108N and 164L) were found in most areas. The mutations 59R and 108N were more frequently found at high proportions as compared to 51I and 164L. The proportions of these mutations ranged from 0 to 65.4% for 59R, 8.3–72.7% for 108N, 0–23.1% for 51I, and 0–21.1% for 164L (Fig. 5a). A total of 15 *pfdhfr* resistance genotypes were found across the areas as single (16.3%), double (25.5%), and triple mutants (7.9%). Genotype richness was seen in Delhi (10 genotypes) and Haryana (12 genotypes) even though a high proportion of *P. falciparum* isolates were wild type (Fig. 5b). The single mutant A_16_C_50_N_51_C_59_**N**_108_I_164_ was mainly found in Uttar Pradesh at a proportion of 55.6%, while the double mutant A_16_C_50_N_51_**R**_59_**N**_108_I_164_ was mostly reported seen Raipur (57.8%) and Goa (60%). The triple mutant A_16_C_50_**I**_51_**R**_59_S_108_**L**_164_ was reported only in Haryana (1.9%) while the double mutant A_16_C_50_N_51_C_59_**N**_108_**L**_164_ was seen only in Jharkhand (4.5%) (Fig. 4b). Interestingly, the richness in resistance genotypes increased over years in Delhi with two, seven and eight genotypes in 1994, 2000 and 2008–2010, respectively. A specific distribution of *pfdhfr* haplotypes between two areas of Orissa (i.e., Rourkela and Bissam Cuttack—BCK) was noted. The mutants A_16_C_50_**I**_51_C_59_S_108_I_164_, A_16_C_50_N_51_C_59_**N**_108_I_164_ and A_16_C_50_N_51_**R**_59_**N**_108_I_164_ were found only in Rourkela while A_16_C_50_**I**_51_C_59_**N**_108_I_164_ and A_16_C_50_**I**_51_C_59_**N**_108_ **L**_164_ were found only in BCK (Fig. 5b). No novel mutation and synonymous mutations were found in *pfdhfr* and *pfdhps* sequences analysed.

**Fig. 6:**
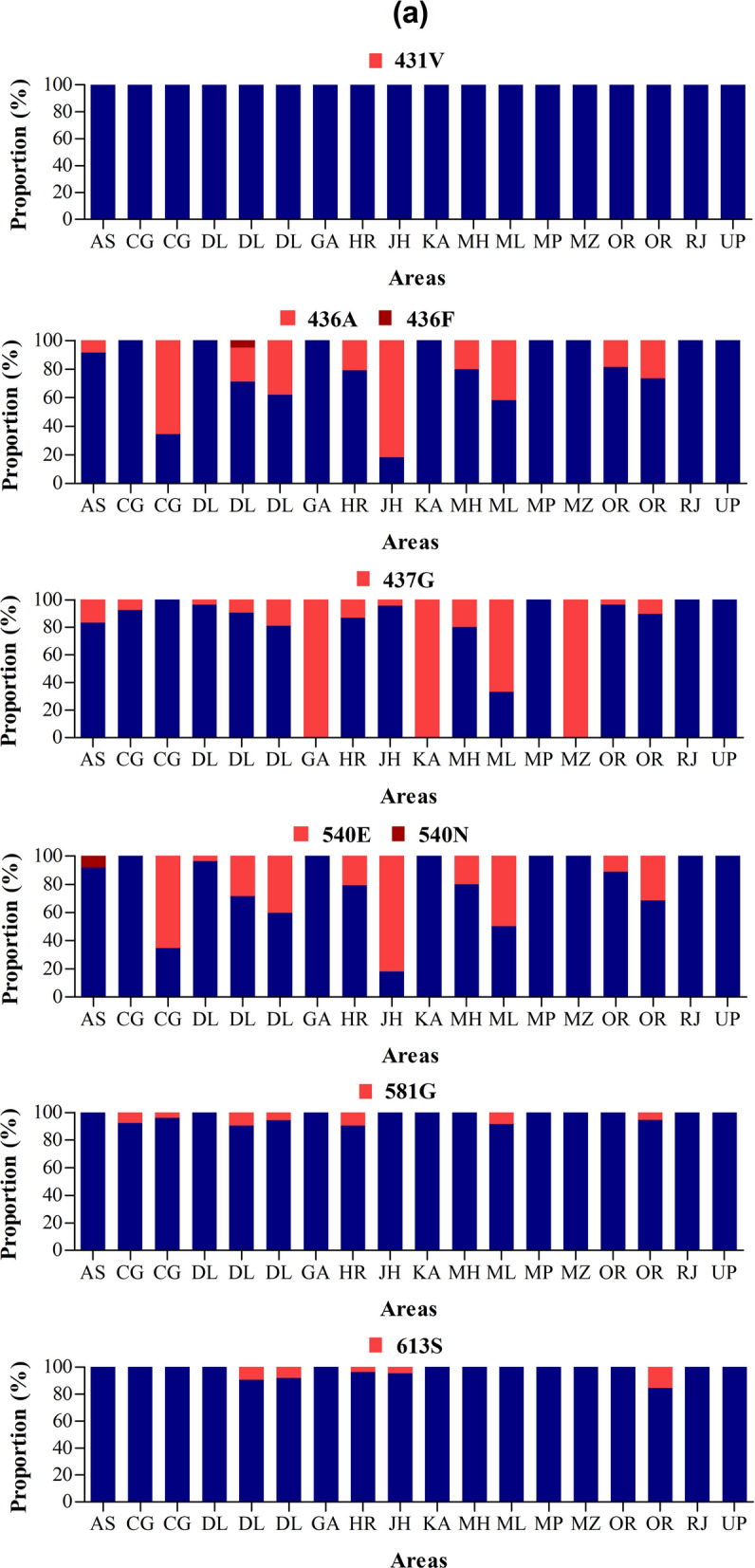

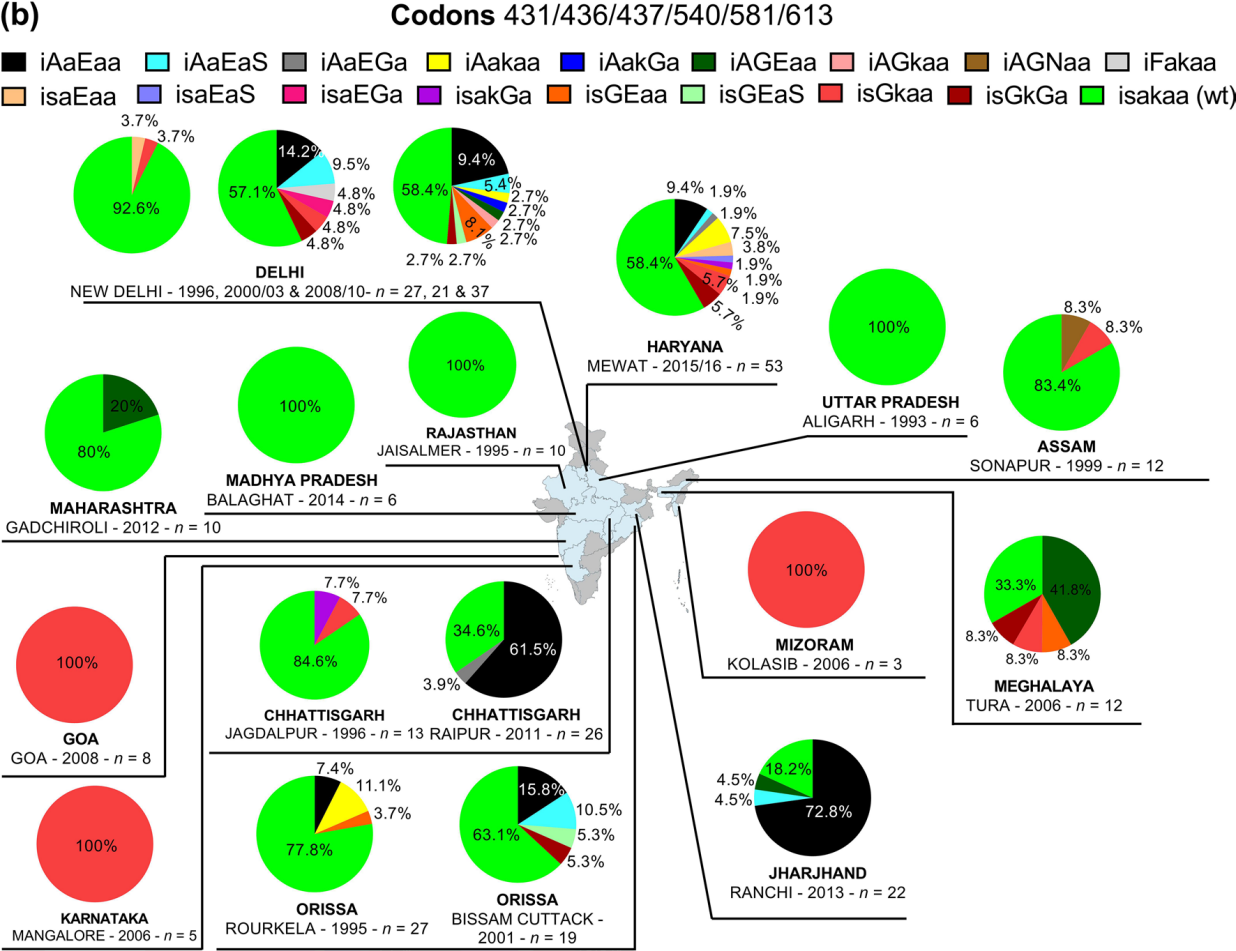
*Pfdhps* genotypes by year and area. *Pf*: *P. falciparum*; *dhps*: dihydropteroate synthase gene; wt: wild type. The international codes of areas were used. *AS* Assam, *CG* Chhattisgarh, *DL* Delhi, *GA* Goa, *HR* Haryana, *JH* Jharkhand, *KA* Karnataka, *MH* Maharashtra, *ML* Meghalaya, *MP* Madhya Pradesh, *MZ* Mizoram, *OR* Orissa, *RJ* Rajasthan, *UP* Uttar Pradesh. In **a**, sample size was different for each area. AS (*n* = 12), CG (*n* = 13 and 26), DL (*n* = 27, 21, and 37), GA (*n* = 8), HR (*n* = 53), JH (*n* = 22), KA (*n* = 5), MH (*n* = 10), ML (*n* = 12), MP (*n* = 6), MZ (*n* = 3), OR (*n* = 27 and 19), RJ (*n* = 10), UP (*n* = 6). **a** Proportion of *pfdhps* 431V, 436A/F, 437G, 540E/N, 581G and 613S mutations, **b** proportion of *pfdhps* haplotypes. In **b** wild alleles are in lower case and mutant alleles are in upper case. Samples from Gautaum Budh Nagar (UP) was excluded from percentage calculation because of low sample size (*n* = 1). The map depicted here is taken from official website of Ministry of External Affairs, Government of India (https://mea.gov.in/india-at-glance.htm, accessed 15/11/2021)

For *pfdhps*, the mutations 436A/F, 437G and 540E were most frequently seen across areas as compared to 431V, 581G and 613S. The proportion of 436A/F mutation varied from 0% (in Goa, Madhya Pradesh, Rajasthan, Uttar Pradesh, and Mizoram) to 81.8% (in Chhattisgarh). All *P. falciparum* isolates from Goa, Karnataka, and Mizoram were carrying the 437G mutation while highest rates of 540E mutation were seen in Jharkhand (81.8%), Chhattisgarh (65.4%) and Meghalaya (50%) (Fig. 6a). Wild type-like haplotype accounted for 59.1% of all isolates, while the rest consisted of single mutants (11.9%), double mutants (22%) and triple mutants (6.9%). A higher genotype richness was found on analysis of *pfdhps* gene compared to *pfhdfr* gene, with 17 resistance genotypes. The highest number of genotypes were found in Delhi in 2000 (7 genotypes), Delhi in 2008/10 (10 genotypes), and Haryana in 2015/16 (11 genotypes) (Fig. 6b). The double mutant I_431_**A**_436_A_437_**E**_540_A_581_A_613_ was most spread as found in five areas at proportion of 14.2–21.6% (Delhi), 9.4% (Haryana), 72.8% (Jharkhand), 61.5% (Chhattisgarh), and 7.4–15.4% (Orissa). The wild type haplotype was found in 100% of *P. falciparum* isolates from Rajasthan, Madhya Pradesh, and Uttar Pradesh, while the double mutant I_431_S_436_**G**_437_K_540_**G**_581_A_613_ was found in 100% of isolates from Goa, Karnataka, and Mizoram (Fig. 5b). The double mutant I_431_S_436_A_437_**E**_540_**G**_581_A_613_ was found only in Delhi (4.8%) while triple mutants I_431_**A**_436_A_437_**E**_540_**G**_581_A_613_ and I_431_**A**_436_**G**_437_**N**_540_A_581_A_613_ were found only in Chhattisgarh (3.9%) and Assam (8.3%), respectively. Five of the nine isolates with triple mutant I_431_**A**_436_**G**_437_**E**_540_A_581_A_613_ were found in Meghalaya.

By combining SP resistance related *pfhdfr* and *pfdhps* mutations, we found 56 *pfhdfr*-*pfhdps* haplotypes represented by single mutants (six types), double mutants (15 types), triple mutants (12 types), quadruple mutants (14 types), quintuple mutants (6 types), and sextuple mutants (3 types) (Additional file 6). The quadruple mutant ACN**RN**I–I**A**A**E**AA accounted for 62.8% (27/43) of all quadruple mutants reported in the study, and was mainly seen in Chhattisgarh (Raipur). Two types of quintuple mutants viz. ACN**RNL**–I**A**A**E**AA and ACN**RN**I–I**A**A**E**A**S** accounted for 42.9% (6/14) and 28.6% (4/14) of all quintuple mutants, and were reported in Delhi, Orissa, and Jharkhand. To be noted, one isolate with quintuple mutant genotype (AC**IRN**I–IS**GE**AA) was found in Delhi. The sextuple mutants consisted of ACN**RNL**–I**A**A**E**A**S** (two isolates), AC**I**C**NL**–I**A**A**E**A**S** (one isolate) and ACI**RNI**–I**A**A**E**A**S** (one isolate); and were found in Delhi, Orissa, and Haryana (Additional file 6).

#### *Pfmdr1*

**Fig. 7:**
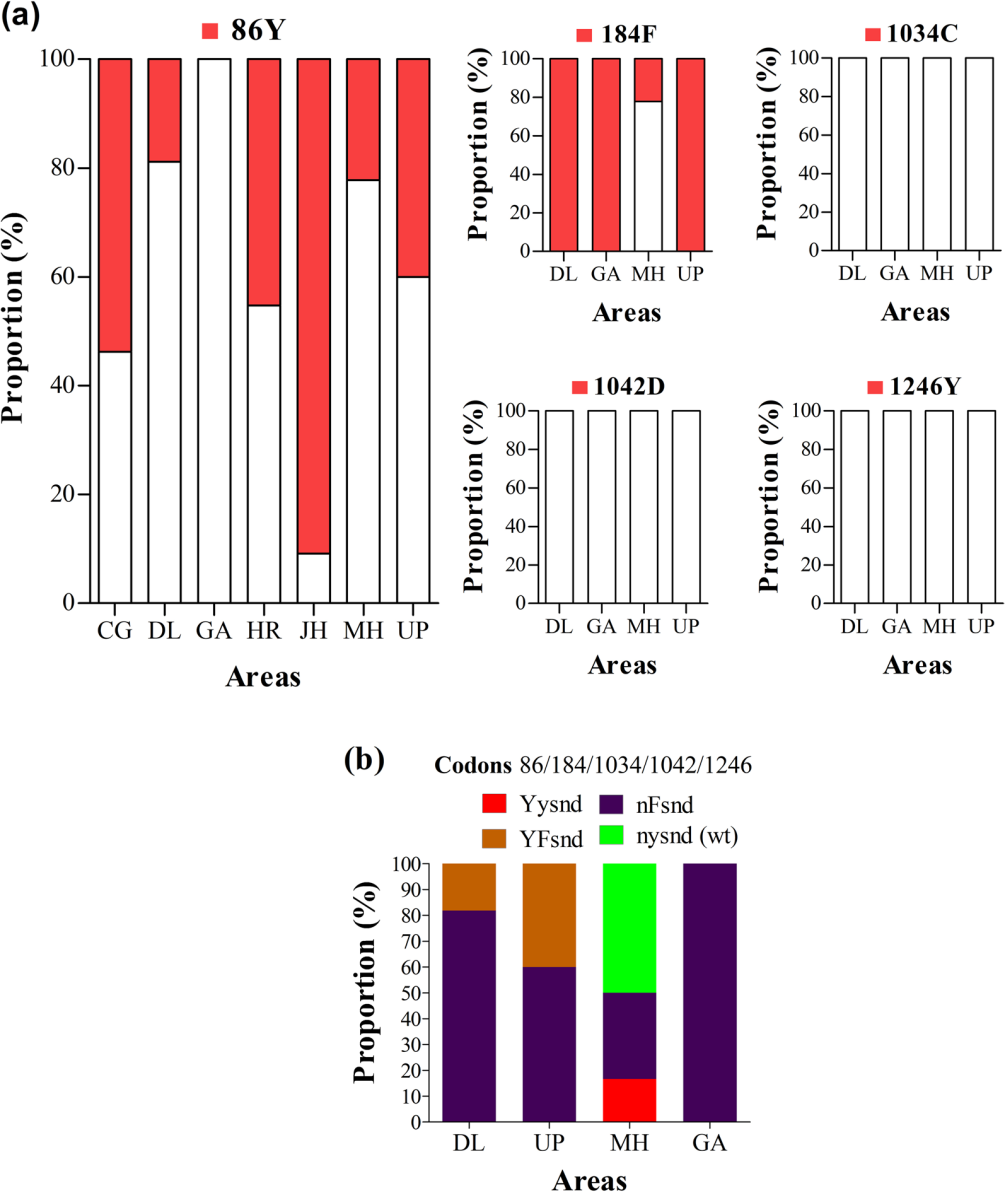
*Pfmdr1* genotypes by year and area. **a** Proportion of *pfmdr1* 86Y, 184F, 1034C, 1042D and 1246Y mutations, **b** proportion of *pfmdr1* haplotypes. *Pf*: *P. falciparum*; *mdr1*: multidrug resistance protein 1 gene; wt: wild type. The international codes of areas were used. *CG* Chhattisgarh, *DL* Delhi, *GA* Goa, *HR* Haryana, *JH* Jharkhand, *MH* Maharashtra, *UP* Uttar Pradesh. In **a**, sample size was different for each area. CG (*n* = 26), DL (*n* = 16), GA (*n* = 4), HR (*n* = 53), JH (*n* = 22), MH (*n* = 9), UP (*n* = 5). In **b** wild alleles are in lower case and mutant alleles are in upper case. The map depicted here is taken from official website of Ministry of External Affairs, Government of India (https://mea.gov.in/india-at-glance.htm, accessed 15/11/2021)

Two of the five resistance *pfmdr1* mutations (i.e., 86Y and 184F) were found in 135 samples from seven regions (Chhattisgarh, Delhi, Goa, Haryana, Jharkhand, Maharashtra, and Uttar Pradesh). The proportion of 86Y and 184F mutations ranged from 0 to 90.9% and 0 to 100%, respectively (Fig. 7a). Three types of mutants were found in this study, and were represented by **Y**_86_Y_184_S_1034_N_1042_D_1246_, N_86_**F**_184_S_1034_N_1042_D_1246_, and **Y**_86_**F**_184_S_1034_N_1042_D_1246_. The double mutant **Y**_86_**F**_184_S_1034_N_1042_D_1246_ was found only in Delhi (18.2%) and Uttar Pradesh (40%). Wild type-like *pfmdr1* isolates were found only in Maharashtra (50%) (Fig. 7b). One synonymous *pfmdr1* mutation (G182G) was found in 25 isolates from Goa (*n* = 4), Maharashtra (*n* = 2), Uttar Pradesh (*n* = 5), and Delhi (*n* = 14).

#### *Pfk13*

In this study, *pfk13* sequences of 90 samples from Haryana (*n* = 53), Orissa (*n* = 8), Uttar Pradesh (*n* = 3), Karnataka (*n* = 4), Madhya Pradesh (*n* = 4) and Delhi (*n* = 18) were analysed. All isolates analysed for mutations in *pfk13* gene were wild type, and no synonymous or nonsynonymous mutations for validated point mutations were observed. No novel mutation and synonymous mutations were found in *pfk13* sequences analysed.

### Profile of *P. vivax* drug resistance genes

#### *Pvcrt-o*

Thirty-two samples from Delhi were analysed in this section. Genetic profiling of *Pvcrt* sequences was available only for samples collected from Delhi in 2017. On analysis, the K10 “AAG” insertion was not detected in any sample.

#### *Pvdhfr* and *pvdhps*

**Fig. 8:**
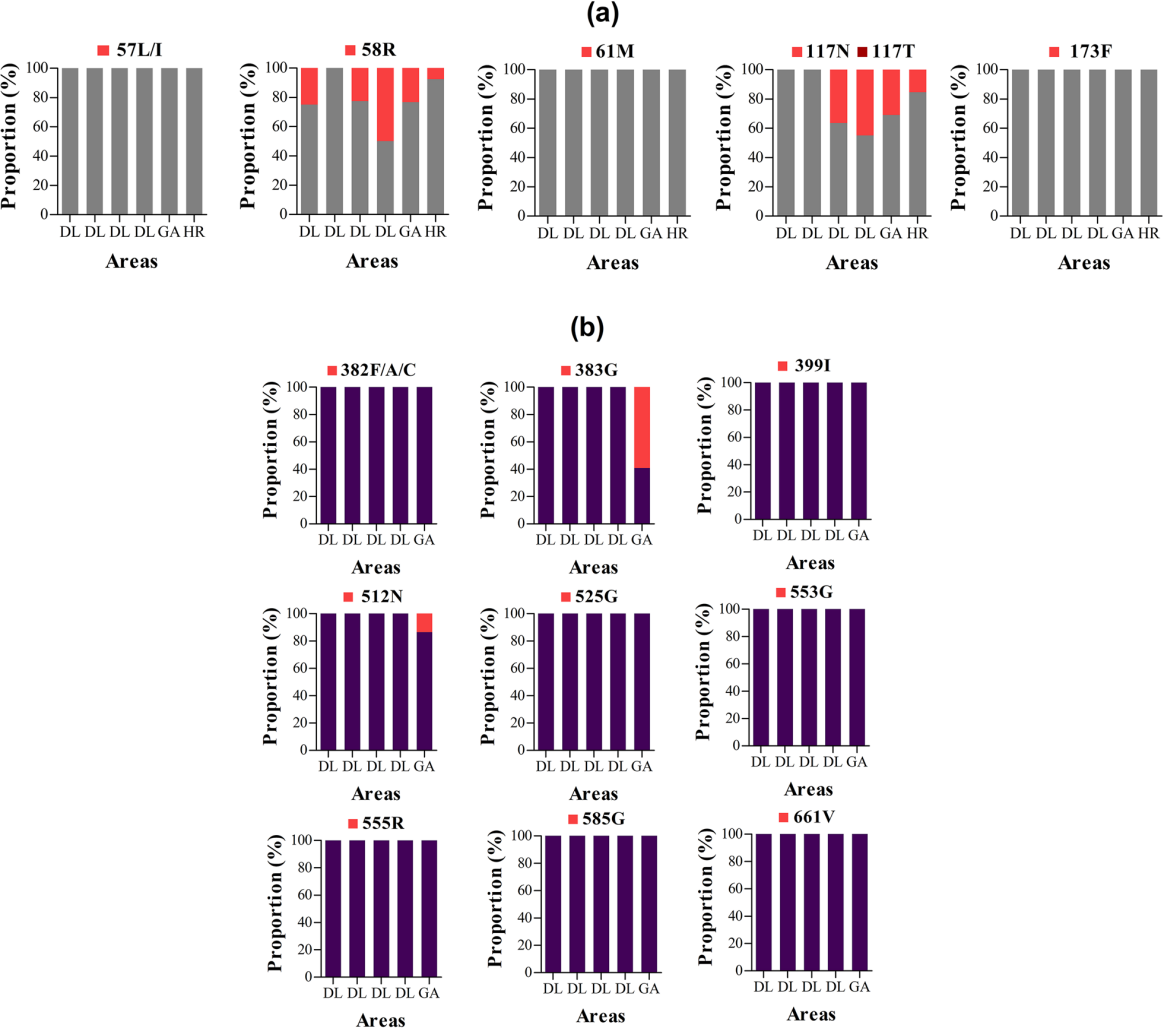

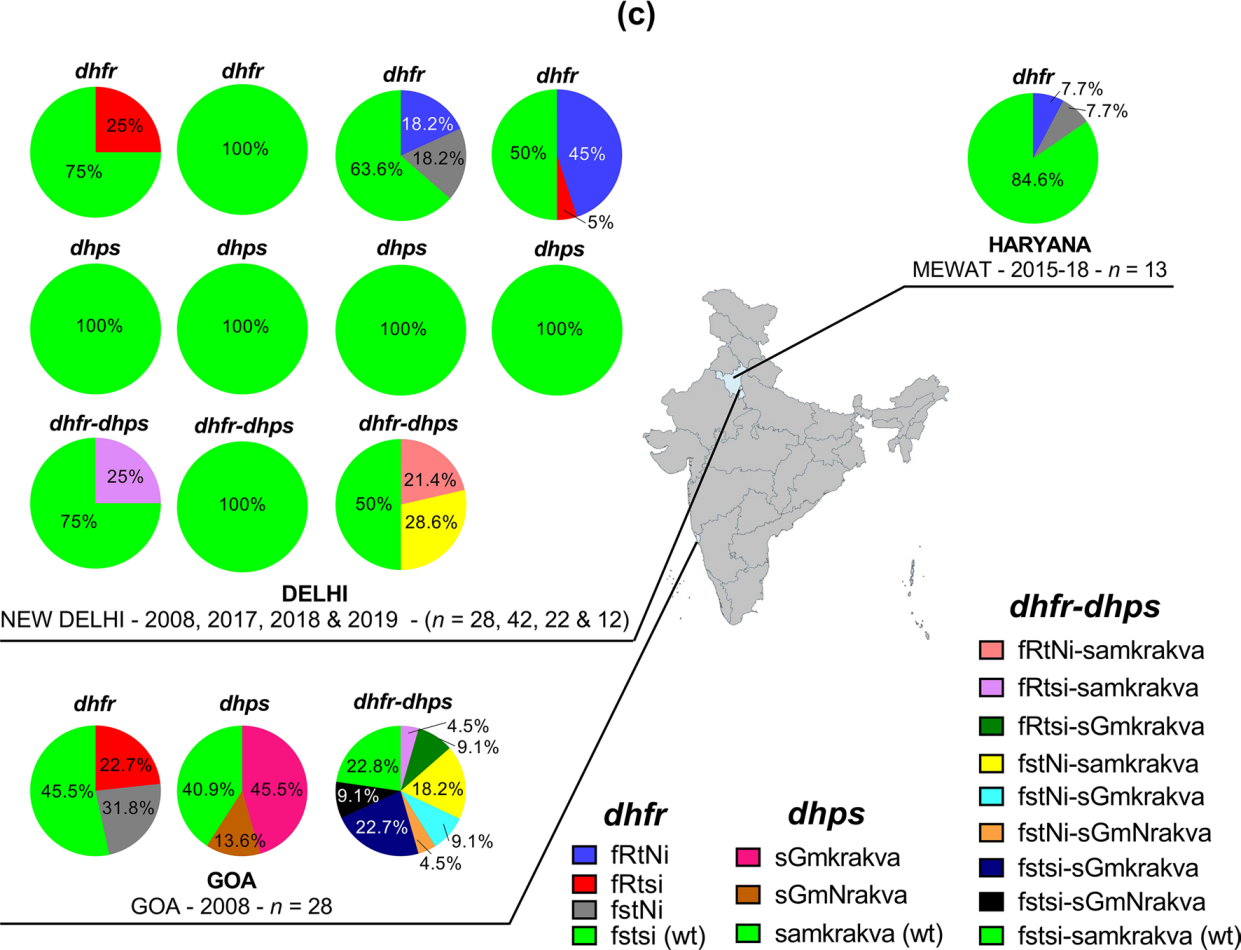
*Pvdhfr* and *Pvdhps* genotypes by year and area. **a** Proportion of *pvdhfr* 57L/I, 58R, 61M, 117N/T and 173F mutations, **b** proportion of *pvdhps* 382F/A/C, 383G, 399I, 512N, 585G, 553G, 555R, 585G, and 661V mutations, **c** proportion of the *pvdhfr* and *pvdhps* haplotypes. *Pv*: *P. vivax*; *dhfr*: dihydrofolate reductase; *dhps*: dihydropteroate synthase; wt: wild type. The international codes of areas were used. *DL* Delhi, *GA* Goa, *HR* Haryana. In **a**, sample size was different for each area. DL (*n* = 8, 32, 22, and 20 in years 2008, 2017, 2018 and 2019), GA (*n* = 22), HR (*n* = 13). In **b**, sample size was different for each area. DL (*n* = 28, 42, 22, and 12 in years 2008, 2017, 2018 and 2019), GA (*n* = 22). In **c** wild alleles are in lower case and mutant alleles are in upper case. The map depicted here is taken from official website of Ministry of External Affairs, Government of India (https://mea.gov.in/india-at-glance.htm, accessed 15/11/2021)

In total, 117 and 126 samples from Delhi, Mewat, and Goa regions were analysed for drug resistance mutations in *pvdhfr* and *pvdhps*, respectively. Of the six *pvdhfr* point mutations analysed in sequences, amino acid changes were detected only in codons 58 and 117, with proportions ranging from 0 to 50% and 0 to 45%, respectively. The proportions of these two mutations were lower in Haryana compared to Delhi, and Goa (Fig. 8a). Similarly, amino changes were detected only in two of the nine *pvdhps* codons analysed namely 383 and 512. Also, all *pvdhps* mutations were found in *P. vivax* isolates from Goa, and were represented by 383G (59.1%) and 512N (13.6%) (Fig. 8b).

The genotype analysis revealed three and two mutated genotypes for *pvdhfr* (F_57_**R**_58_T_61_S_117_I_173_, F_57_S_58_T_61_**N**_117_I_173_ and F_57_**R**_58_T_61_**N**_117_I_173_) and *pvdhps* (S_382_**G**_383_M_399_K_512_R_525_A_553_K_555_V_585_A_661_ and S_382_**G**_383_M_399_**N**_512_R_525_A_553_K_555_V_585_A_661_), respectively (Fig. 8c). Wild type isolates accounted for 89.6% of all *pvdhps* genotypes.

The combination of *pvdhfr*–*pvdhps* genotypes revealed nine haplotypes where most were represented by wild type. No mutant *pvdhfr*–*pvdhps* haplotypes were found in *P. vivax* isolates from Delhi collected in 2017, but single mutant FST**N**I–SAMKRAKVA (28.6%) and double mutant F**R**T**N**I-SAMKRAKVA (21.4%) were found in *P. vivax* isolates collected in 2018. In Goa, mutants were mainly represented by FSTSI–S**G**MKRAKVA (16.7%), FST**N**I–SAMKRAKVA (13.3%) and F**R**TSI–SAMKRAKVA (10%) (Fig. 7c). No novel mutation and synonymous mutations were found in *pvdhfr* and *pvdhps* sequences analysed.

#### *Pvmdr1*

**Fig. 9:**
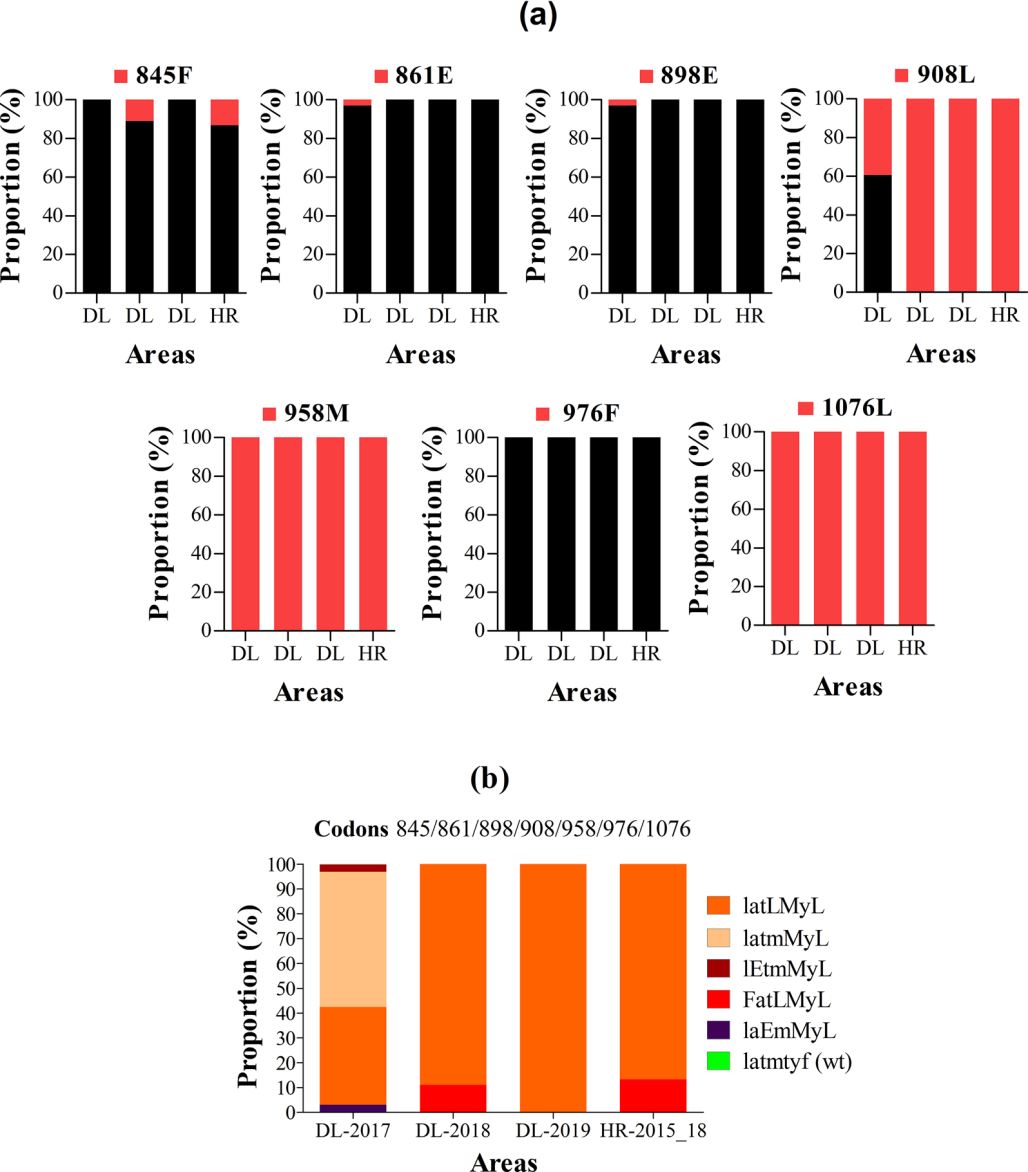
*Pvmdr1* genotypes by year and area. **a** Proportion of *pvmdr1* 845F, 861E, 898E, 908L, 958M, 976F and 1076L mutations, **b** proportion of *pvmdr1* haplotypes. *Pv*: *P. vivax*; *mdr1*: multidrug resistance protein 1, wt: wild type. The international codes of areas were used. *DL* Delhi, *HR* Haryana. In **a**, sample size was different for each area. DL (*n* = 33, 18 and 24 in years 2017, 2018 and 2019), HR (*n* = 15). In **b** wild alleles are in lower case and mutant alleles are in upper case. The map depicted here is taken from official website of Ministry of External Affairs, Government of India (https://mea.gov.in/india-at-glance.htm, accessed 15/11/2021)

On analysis of 90 nucleotide sequences for *pvmdr1* gene, amino acid changes were detected at all codons analysed with the exception of codon 976. The 845F, 861E and 898E were least frequently found mutations in *P. vivax* isolates with proportions of 0–13.3%, 0–3%, and 0–3%, respectively. The 908L mutation were at proportion of 39.4–100% in Delhi and 100% in Haryana. All *P. vivax* isolates carried 958M and 1076L mutations (Fig. 9a). No novel mutation and synonymous mutations were found in *pvmdr1* sequences analysed.

The genotype analysis pointed out the absence of wild type L_845_A_861_T_898_M_908_T_958_Y_976_F_1076_ in all areas. Five *pvmdr1* genotypes were reported in this study, and were predominantly represented by triple mutant LAT**LM**Y**L** in isolates from Haryana (86.7%) and isolates from Delhi collected in 2018 (88.9%) and 2019 (100%). In contrast, double mutant LATM**M**Y**L** accounted for 54.5% of all mutants found in isolates from Delhi collected in 2017. One quadruple mutant **F**AT**LM**Y**L** was found in Delhi—2018 (11.1%) and Haryana (13.3%) (Fig. 9b).

#### *Pvk12*

The analysis of 30 *pvk12* sequences from Delhi and Goa regions revealed no mutations at codons 88, 124, 552, 581 and 697. Two novel mutations K264R (nonsynonymous) and L617L (synonymous) were found in Goa samples at proportions of in 27.3% (6/22) and 4.5% (1/22), respectively.

## Discussion

The present study aimed at delineating genetic profile of main genes associated with drug resistance in *P. falciparum* and *P. vivax* malaria over 30 years in India.

### Profile of anti-malarial drug resistance *P. falciparum* genes

#### *Pfcrt*

Most of *pfcrt* genotypes in study areas were double mutant **S**VMN**T** due to high rates of single mutations 72S and 76T, and this finding corroborates with previous reports [41–43]. In contrast, other studies reported lower rates of this double mutant in Odisha, and Arunachal Pradesh [44, 45]. A detailed analysis of the nucleotide codon at position 72 revealed that all **S**VMN**T** mutants from this study were of type S_(agt)_VMNT reported to have originated from Papua New Guinea [46]. The triple mutant CV**IET** was predominantly observed only in Maharashtra, which is not in line with previous reports from the same state [41]. The triple mutant was also reported from other states such as Odisha, Arunachal Pradesh, Chhattisgarh, and Assam [41, 43–45]. Area and time related drug policy changes could explain these between-study differences of **S**VMN**T** and CV**IET** proportions. The epidemiological profile of *pfcrt* genotypes hugely varies outside India, where triple mutant CV**IET** is predominant in countries, such as Cameroon and Saudi Arabia [47, 48], while wild type CVMNK is predominant in Ethiopia, Malawi, and Tanzania [49–51].

#### *Pfdhfr* and *pfdhps*

SP has been adopted and implemented in India for treating malaria cases in 1995. The frequent *pfdhfr* and *pfdhps* mutants were reported in Delhi in 1994 (e.g. A_16_C_50_**I**_51_C_59_**N**_108_I_164_) and Odisha in 1995 (I_431_**A**_436_A_437_**E**_540_A_581_A_613_, I_431_S_436_**G**_437_**E**_540_A_581_A_613_ and I_431_**A**_436_A_437_K_540_A_581_A_613_). These findings indicate that these current validated mutations associated with SP resistance were already present before 1995. This result could be likely due to drug pressure exerted by SLP drug which was prescribed in country till 1995 (Fig. 1).

Double mutations in *pfdhfr* were predominant in this study, and this finding is consistent with that of previous systematic review that double *pfdhfr* mutations are dominant in India with overall proportion of 57% [25]. Also, we found that R_59_N_108_ double mutation was most frequently seen in all *pfdhfr* double and triple mutants reported in present study. Only R_59_N_108_L_164_ and I_51_R_59_N_108_ triple mutants were reported in India so far [52]. Two new triple mutants (i.e., I_51_N_108_L_164_ and I_51_R_59_L_164_) were found in *P. falciparum* isolates from Mewat (Haryana).

Regarding *pfdhps*, double mutations were most frequently seen in contrast to other reports that found a predominance of triple mutations in India [25]. Due to high circulation of SP-resistant *P. falciparum* populations in NE states (Assam, Mizoram, Meghalaya, Manipur, Nagaland, Tripura, Arunachal Pradesh), AS + SP has been replaced by AL as current malaria treatment [53–55]. The *pfdhps* A_436_G_437_E_540_ triple mutations are highly prevalent in these states, and the findings from the study support this fact as 55.6% of isolates carrying these three mutations were seen in Meghalaya samples. It is noteworthy that another triple mutation (A_436_G_437_N_540_) was found in *P. falciparum* isolates collected from Assam in the year 1999. The first description of this triple mutant in India was documented in 2005 in isolates from The Nicobar Islands [56]. Thus, the present study confirms that this A_436_G_437_N_540_ triple mutant was circulating in India before 2005.

Finally, this study reports high rates (34.7%) of *pfdhfr*-*pfdhps* quadruple mutants but quintuple and sextuple mutants at fewer rates (11.3% and 3.2%). One isolate from Delhi showed quintuple mutations, I_51_R_59_N_108_–G_437_E_540_, associated with fully resistance to SP, and strong predictor of clinical SP treatment failure [57, 58]. Imported malaria and SLP/SP drug pressure could likely explain such high rates of drug resistance mutants in *pfdhfr*/*pfdhps*. SLP, a sulfamide analogue is used 1982 in India for treatment of CQ-resistant *P. falciparum* malaria based on satisfactory evidence from clinical studies [59], while SP was implemented 13 years later. Also, most *pfdhfr*/*pfdhps* samples (70.3%) analysed were collected in 1994 and 1995, and drug pressure exerted by SLP through cross-resistance mechanism could explain drug resistance mutations found in these samples collected before official implementation of SP. Finally, SP pressure and human migration are also additional determinants of drug resistance in *pfdhfr*/*pfdhps* samples by exerting drug pressure and bringing mutant alleles from other countries, respectively.

#### *Pfmdr1*

The *pfmdr1* SNPs at 86Y and 184F were found in the isolates at varying proportions in study sites consistent with previous studies that reported high proportions of *pfmdr1* 86Y mutation in West Bengal, Chhattisgarh, and Odisha [42–44], but contradicting findings were reported from Mizoram, Meghalaya, and Tripura [60]. In general, the 86Y and 184F mutations are more commonly seen in Asian and African settings, while the 1034C, 1042D and 1246Y mutations are more frequent in South America settings [61]. There is strong link between *pfmdr1* 86Y mutation and acquisition of resistance phenotype to CQ and AQ, while sensitivity phenotype to DHA, MEF and L [61]. Evidence for involvement of 184F mutation is still limited [61, 62].

#### *Pfk13*

No mutations associated with ART-resistance were found in this study. This is in line with previous studies, even though four ART-resistance validated mutations (i.e., 446I, 539T, 561H, 625R) have been reported at very marginal proportions in two areas of India (Arunanchal Pradesh, West Bengal) [63–65]. Other *pfk13* polymorphisms (189T, 481V, 533A, 549Y, 578S, 579T, 657H, 672S, 675V, and 702N) have been also reported in these two regions and elsewhere (Mizoram, Tripura, Madhya Pradesh, and Assam) [63, 64, 66–68]. This implies that ART-resistance has not yet emerged, but molecular surveillance should be continuously carried out in malarious regions in India.

### Profile of anti-malarial drug resistance *P. vivax* genes

#### *Pvcrt-o*

Previous in vitro studies reported link between the *pvcrt-o* K10 insertion and decreased susceptibility of *P. vivax* parasites to CQ [38], thereby suggesting possible role of this mutation in modulating *P. vivax* susceptibility. In the present study, all isolates were wild type consistent with previous reports from Thai–Cambodian border, Thailand and China–Myanmar border [69–71]. However, this finding is not consistent with that of other studies conducted in India which reported K10 proportion of ~ 9.5–17.5% and 5.6% in Chandigarh (North India) and Mangalore (South India), respectively [72, 73]. Likewise, higher proportions were reported in other endemic regions such as Myanmar (~ 28.2–72.7%) and China–Myanmar border (33.2%) [74–76]. All these findings indicate a spatiotemporal variation of K10 proportion in *P. vivax* areas.

#### *Pvdhfr* and *pvdhps*

Double and triple/single mutants in *pvdhps* accounted for ~ 33% and ~ 60% of all mutants across India [25]. In this study, double and single mutants were found at overall proportion of 11.9% and 17%, respectively. Double mutants R_58_N_117_ were predominant in this study, especially in Delhi, as also previously reported from southern and western parts of India (i.e., Tamil Nadu, Karnataka, and West Bengal) [77–79]. This finding is also consistent with other *P. vivax* endemic countries such as China–Myanmar border, Ethiopia, and Sudan [75, 80, 81]. Imwong and colleagues showed the *pvdhfr* R_58_ and N_117_ mutations are the first to appear when drug pressure is applied [82], and this could likely explain high rates of these mutations in India where SP pressure is high due to utilization of the ACT AS + SP as nationwide first-line treatment of uncomplicated malaria with the exception of NE states. No triple and quadruple mutants were found in this study, which is consistent with previous studies in India that indicates probably a focused geographical distribution of these mutants in NE states of the country [25]. On analysis of the *pvdhps* gene, the wild type S_382_A_383_M_399_K_512_R_525_A_553_K_555_V_585_A_661_ was predominantly seen in *P. vivax* isolates, and this is line with the current situation on *pvdhps* genetic profile in India [25]. Only two types of *pvdhps* mutants were reported in the present study (S**G**KAV and S**GN**AV), and these were also reported in earlier reports from Delhi, West Bengal, Karnataka, Rajasthan, and Tamil Nadu [25]. For the first time this study reports the presence of double mutant S**GN**AV in Goa samples  from India.

#### *Pvmdr1*

The *pvmdr1* 908L, 958M and 1076L mutations were highly prevalent in *P. vivax* samples with proportion of 100% in most study areas. Such findings were also reported previously in India, Ethiopia, Pakistan, and China–Myanmar border, but much lower proportions were reported from Thailand [69, 70, 72, 73, 76, 83, 84]. The 976F mutation, found associated with CQ resistance in vitro [38], was not found among *P. vivax* isolates and this supports earlier findings of low prevalence of this mutation from settings, such as India (7%) and China–Myanmar border (~ 0–2.7%) [70, 72, 73, 75], but contrasting with those from Thailand (~ 1.7–26.7%) and Indonesia (~ 66.7–96.1%) [69, 74, 85].

#### *Pvk12*

To the best knowledge of authors, this is first study on *pvk12* polymorphism from India. A limited polymorphism was found in *pvk12*, the *P. vivax* orthologue gene of *pfk13*, with one novel nonsynonymous polymorphism (K264R) in isolates from Goa. Other nonsynonymous mutations have been reported from SEA and Oceania areas namely N57I, M124I, S452R, R501K, V541A, E553K, C566G (China–Myanmar border), I537V (Vanuatu), V552I (Cambodia, Malaysia), M548I (Thai–Cambodian border), G581R (China), K596R and P641L (Thai–Cambodian border), and V652L (Solomon Islands) [71, 86–91]. In contrast, other studies from SEA (China–Myanmar border) and Africa (Mauritania) reported no *pvk12* polymorphism in *P. vivax* isolates collected from local and imported malaria patients [76, 92, 93]. It is still elusive if these mutations within and outside the *pvk12* propeller domain can modulate *P. vivax* susceptibility to ACT, thereby requiring further research.

## Limitations

This study should be interpreted in light of its limitations. First, samples were not from all Indian regions and this limits the representativeness of results at national level. Second, not all samples could be sequenced analysed in this study. Finally, due to low number of good quality sequences for some study sites and year of sample collection, it was impossible to apply sophisticated statistical methods such as generalized equation models to analyse the evolution of resistance mutation over time.

## Conclusions

The profiling of genetic markers associated with *P. falciparum* and *P. vivax* drug resistance was determined over a 30-year timeframe in India. The analysis revealed substantial spatiotemporal changes with increase in SNPs related to genetic profile of anti-malarial drug molecular markers in *P. falciparum* and *P. vivax* populations over 30 years. These findings support continuous surveillance and characterization of *P. falciparum* and *P. vivax* populations as proxy of the effectiveness of anti-malarial drugs in India.

### Supplementary Information

**Additional file 1.** Source of *P. falciparum* and *P. vivax* samples analysed in the study.

**Additional file 2.** Details of urbanization and malaria endemicity level in study areas.

**Additional file 3.** Primers and PCR conditions of the *P. falciparum* and *P. vivax 18sRNA* and anti-malarial drug resistance genes.

**Additional file 4.** Flow diagram showing number of samples analysed in the study for *P. falciparum* and *P. vivax* drug resistance genes.

**Additional file 5.** Electrophoresis gels of different *P. falciparum* and *P. vivax* drug resistance gene amplicons.

**Additional file 6.** Resistance haplotypes by combining *pfdhfr* (codons 16, 50, 51, 59, 108, 164) and *pfdhps* (codons 431, 436, 437, 540, 581, 613).

## Data Availability

All the data supporting the study findings are within the manuscript. Additional detailed information and raw data will be shared upon request addressed to the corresponding author.

## Abbreviations

ACT: Artemisinin based combination therapy
AL: Artemether–lumefantrine
AQ: Amodiaquine
ART: Artemisinin
AS: Artesunate
*crt*: Chloroquine resistant transporter gene
CQ: Chloroquine
*dhfr*: Dihydrofolate reductase gene
*dhps*: Dihydropteroate synthase gene
*mdr1*: Multidrug resistance protein 1
MQ: Mefloquine
N/A: Not applicable
NCBI: National Center for Biotechnology Information
NE: North east states
PCR: Polymerase chain reaction
PG: Proguanil
PPQ: Piperaquine
PQ: Primaquine
QN: Quinine
Q.S: *Quamtum satis*
SEA: Southeast Asia
SLP: Sulfalene–pyrimethamine
SP: Sulfadoxine–pyrimethamine
WHO: World Health Organization
wt: Wild type
