# Supplementary material for: Nationwide spatiotemporal drug resistance genetic profiling from over three decades in Indian Plasmodium falciparum and Plasmodium vivax isolates
Source: Malar J. 2023 Aug 15;22:236. doi: 10.1186/s12936-023-04651-x (PMC10428610; doi:10.1186/s12936-023-04651-x)
Supplement: Supplementary file 1 — Additional file 1. Source of P. falciparum and P. vivax samples analysed in the study. [file 12936_2023_4651_MOESM1_ESM.docx]

**Additional file 1**. Source of *P. falciparum* and *P. vivax* samples analysed in the study

***Plasmodium falciparum***

| **Areas** | **State/Union territory** | **Year of sample collection** | **Source of samples** | **Malaria diagnosis methods** | **Type of malaria** |
| --- | --- | --- | --- | --- | --- |
| Allahabad | Uttar Pradesh | 1993 | Parasite Bank | LM + RDT | Symptomatic |
| Balaghat | Madhya Pradesh | 2014 | Parasite Bank | LM + RDT | Symptomatic |
| Bissum & Cuttack | Odisha | 2001 | Field + Clinic | LM + RDT | Symptomatic + Asymptomatic |
| Gadchiroli | Maharashtra | 2012 | Parasite Bank | LM + RDT | Symptomatic |
| Gautam Budh Nagar | Uttar Pradesh | 2000 | Parasite Bank | LM + RDT | Symptomatic |
| Jagdalpur | Chhattisgarh | 1996 | Field | LM + RDT | Symptomatic + Asymptomatic |
| Jaisalmer | Rajasthan | 1995 | Parasite Bank | LM + RDT | Symptomatic |
| Kolasib | Mizoram | 2007 | Parasite Bank | LM + RDT | Symptomatic |
| Mangalore | Karnataka | 2006 | Parasite Bank | LM + RDT | Symptomatic |
| Mewat | Haryana | 2015  2016  2018 | Clinic  Clinic  Clinic | LM + RDT | Symptomatic |
| New Delhi | Delhi | 1994  2000  2002  2003  2008  2010  2019 | Parasite Bank  Clinic  Parasite Bank  Parasite Bank  Clinic  Clinic  Clinic | LM + RDT | Symptomatic + Asymptomatic |
| Panaji | Goa | 2008 | Parasite Bank | LM + RDT | Symptomatic |
| Raipur | Chhattisgarh | 2011 | Clinic | LM + RDT |  |
| Ranchi | Jharkhand | 2013 | Clinic | LM + RDT |  |
| Rourkela | Odisha | 1995 | Parasite Bank | LM + RDT | Symptomatic |
| Sonapur | Assam | 1999  2006  2007 | Parasite Bank  Field + PHCs  Field + PHCs | LM + RDT | Symptomatic + Asymptomatic |
| Tura | Meghalaya | 2006 | Parasite Bank | LM + RDT | Symptomatic |

LM : Light microscopy, RDT : Rapid diagnostic test

***Plasmodium vivax***

| **Areas** | **State/Union territory** | **Year of sample collection** | **Source of samples** | **Malaria diagnosis methods** | **Type of malaria** |
| --- | --- | --- | --- | --- | --- |
| Aligarh | Uttar Pradesh | 2011 | Parasite Bank | LM + RDT | Symptomatic |
| Bissam Cuttack | Orissa | 2011 | Parasite Bank | LM + RDT | Symptomatic |
| Goa | Goa | 2008 | Parasite Bank | LM + RDT | Symptomatic |
| Jaisalmer | Rajasthan | 2010 | Parasite Bank | LM + RDT | Symptomatic |
| Mangalore | Karnataka | 2006  2008  2009  2013  2015 | Parasite Bank  Parasite Bank  Parasite Bank  Parasite Bank  Parasite Bank | LM + RDT | Symptomatic |
| Mewat | Haryana | 2015  2016  2018 | Field  Field  Field | LM + RDT | Symptomatic + Asymptomatic |
| Mirzapur | Uttar Pradesh | 1996 | Parasite Bank | LM + RDT | Symptomatic |
| New Delhi | Delhi | 1999  2001  2008  2017  2018  2019 | Parasite Bank  Parasite Bank  Parasite Bank  Safdarjung hospital and NIMR clinic  Safdarjung hospital and NIMR clinic  Safdarjung hospital and NIMR clinic | LM + RDT | Symptomatic + Asymptomatic |
| Raipur | Chhattisgarh | 2013  2014  2016 | Parasite Bank  Parasite Bank  Parasite Bank | LM + RDT | Symptomatic |
| Rajasthan | Rajasthan | 2012 | Parasite Bank | LM + RDT | Symptomatic |
| Rourkela | Orissa | 1995  2008  2009  2010 | Parasite Bank  Parasite Bank  Parasite Bank  Parasite Bank | LM + RDT | Symptomatic |
| Shankargarh | Uttar Pradesh | 1996 | Parasite Bank | LM + RDT | Symptomatic |

LM : Light microscopy, RDT : Rapid diagnostic test
