# Supplementary material for: Nationwide spatiotemporal drug resistance genetic profiling from over three decades in Indian Plasmodium falciparum and Plasmodium vivax isolates
Source: Malar J. 2023 Aug 15;22:236. doi: 10.1186/s12936-023-04651-x (PMC10428610; doi:10.1186/s12936-023-04651-x)
Supplement: Supplementary file 2 — Additional file 2. Details of urbanization and malaria endemicity level in study areas. [file 12936_2023_4651_MOESM2_ESM.docx]

**Additional file 2**. Details of urbanization and malaria endemicity level in study areas

| **Areas** | **State/UT** | **Year of sample collection (No. of samples)** | **Dominant *Plasmodium* species at the moment of sample collection** | **Current dominant *Plasmodium* species** | **Current level of malaria endemicity (API)** | **Level of urbanization** |
| --- | --- | --- | --- | --- | --- | --- |
| Aligarh | Uttar Pradesh | 2011 | *Pv* [1–3] | *Pv* [4,5] | < 1 | Rural [6] |
| Allahabad | Uttar Pradesh | 1993 | na | *Pv* [4,5] | < 1 | na |
| Balaghat | Madhya Pradesh | 2014 | *Pv* [1,3,7] | *Pf* [4,5] | < 1 | Rural [6] |
| Bissum & Cuttack | Odisha | 2001  2011 | *Pf* [8]  *Pf* [1–3] | *Pf* [4,5]  *Pf* [4,5] | > 2 – 5 | Rural[9]  Rural [6] |
| Gadchiroli | Maharashtra | 2012 | *Pv* [2,3,7] | *Pf* [4,5] | 1 – 2 | Rural [6] |
| Gautam Budh Nagar | Uttar Pradesh | 2000 | na | *Pv* [4,5] | < 1 | na |
| Jagdalpur | Chhattisgarh | 1996 | na | *Pf* [4,5] | > 5 – 10 | na |
| Jaisalmer | Rajasthan | 1995  2010 | na  *Pv* [1–3] | *Pv* [4,5]  *Pv* [4,5] | < 1 | na  na |
| Rajasthan | Rajasthan | 2012 | *Pv* [1–3] | *Pv* [4,5] | < 1 | Rural [6] |
| Kolasib | Mizoram | 2007 | *Pf* | *Pf* [4,5] | > 10 | Urban [9] |
| Mangalore | Karnataka | 2006  2008  2009  2013  2015 | *Pv* [10]  *Pv* [11,12]  *Pv* [11]  *Pv* [1–3]  *Pv* [1] | *Pv* [4,5]  *Pv* [4,5]  *Pv* [4,5]  *Pv* [4,5]  *Pv* [4,5] | < 1 | na  na  na  Rural [6]  Rural [6] |
| Mirzapur | Uttar Pradesh | 1996 | na | *Pv* [4,5] | < 1 | na |
| Shankargarh | Uttar Pradesh | 1996 | na | *Pv* [4,5] | < 1 | na |
| Mewat | Haryana | 2015  2016  2018 | *Pv* [1]  *Pv* [13]  *Pv* [5] | *Pv* [4,5]  *Pv* [4,5]  *Pv* [4,5] | < 1 | Rural [6] Rural [6]  Rural [6] |
| New Delhi | Delhi | 1994  1999  2000  2001  2002  2003  2008  2010  2017  2018  2019 | na  na  na  na  *Pv* [10]  *Pv* [10]  *Pv* [12]  *Pv* [10]  *Pv* [14]  *Pv* [15]  *Pv* [5] | *Pv* [4,5]  *Pv* [4,5]  *Pv* [4,5]  *Pv* [4,5]  *Pv* [4,5]  *Pv* [4,5]  *Pv* [4,5]  *Pv* [4,5]  *Pv* [4,5]  *Pv* [4,5]  *Pv* [4,5] | > 2 – 5 | na  na  na  Urban[9]  Urban [9]  Urban [9]  Urban [9]  Urban [9]  Urban [6]  Urban [6]  Urban [6] |
| Panaji | Goa | 2008 | Pv [11,12] | *Pf* [4,5] | < 1 | Urban[9] |
| Raipur | Chhattisgarh | 2011  2013  2014  2016 | *Pf* [2,3,11]  *Pf* [1–3]  *Pf* [1–3]  *Pf* [13] | *Pf* [4,5]  *Pf* [4,5]  *Pf* [4,5]  *Pf* [4,5] | > 2 – 5 | Rural [6]  Rural [6]  Rural [6]  Rural [6] |
| Ranchi | Jharkhand | 2013 | Pv [1–3] | *Pf* [4,5] | > 2 – 5 | Rural [6] |
| Rourkela | Odisha | 1995  2008  2009  2010 | na  *Pf* [11]  *Pf* [11]  *Pf* [2,3,11] | *Pf* [4,5]  *Pf* [4,5]  *Pf* [4,5]  *Pf* [4,5] | > 2 – 5 | na  Rural [9]  Rural [9]  Rural [9] |
| Sonapur | Assam | 1999  2006  2007 | *Pf* [16]  *Pf* [17]  *Pf* [17] | *Pv* [4,5]  *Pv* [4,5]  *Pv* [4,5] | < 1 | na  Rural [9]  Rural [9] |
| Tura | Meghalaya | 2006 | *Pf* [18] | *Pf* [4,5] | 1 – 2 | Rural [9] |

**References used**

[1] NVBDCP: Malaria Situation 2011-2015 n.d.

[2] NVBDCP; Malaria Situation 2010-2014 (b) n.d.

[3] NVBDCP; Malaria Situation 2010-2014 (a) n.d.

[4] NVBDCP: Malaria Situation 2021-2022 n.d.

[5] NVBDCP: Malaria situation 2018-2022 n.d.

[6] Census India 2011 - Population of India n.d. https://www.censusindia2011.com/ (accessed September 19, 2022).

[7] Malaria Situation in India from 2014 :: National Center for Vector Borne Diseases Control (NCVBDC) n.d. https://nvbdcp.gov.in/index4.php?lang=1&level=0&linkid=564&lid=3867 (accessed September 15, 2022).

[8] Sahu SS, Gunasekaran K, Vanamail P, Jambulingam P. Persistent foci of falciparum malaria among tribes over two decades in Koraput district of Odisha State, India. Malar J 2013;12:72. https://doi.org/10.1186/1475-2875-12-72.

[9] T C, P G. "Trends and Pattern of Urbanization in India: An Inter State Analysis ". Artha - Journal of Social Sciences 2009;8:9. https://doi.org/10.12724/AJSS.14.2.

[10] NVBDCP: Malaria Situation 2002-2006 n.d.

[11] NVBDCP: Malaria Situation 2008-2012 n.d.

[12] Annual Epidemiological Situation Report 2008 n.d.

[13] NVBDCP: Malaria situation 2016-2020 n.d.

[14] NVBDCP: Annual-Report-2017.pdf n.d.

[15] NVBDCP: Annual-Report-2018.pdf n.d.

[16] Dev V, Phookan S, Sharma VP, Dash AP, Anand SP. Malaria parasite burden and treatment seeking behavior in ethnic communities of Assam, Northeastern India. J Infect 2006;52:131–9. https://doi.org/10.1016/J.JINF.2005.02.033.

[17] Sharma VP, Dev V. Prospects of Malaria Control in Northeastern India with Particular Reference to Assam n.d.

[18] Dev V, Sangma BM, Dash AP. Persistent transmission of malaria in Garo hills of Meghalaya bordering Bangladesh, north-east India. Malar J 2010;9:1–7. https://doi.org/10.1186/1475-2875-9-263/TABLES/4.
