# Supplementary material for: Nationwide spatiotemporal drug resistance genetic profiling from over three decades in Indian Plasmodium falciparum and Plasmodium vivax isolates
Source: Malar J. 2023 Aug 15;22:236. doi: 10.1186/s12936-023-04651-x (PMC10428610; doi:10.1186/s12936-023-04651-x)
Supplement: Supplementary file 3 — Additional file 3. Primers and PCR conditions of the P. falciparum and P. vivax 18sRNA and anti-malarial drug resistance genes. [file 12936_2023_4651_MOESM3_ESM.docx]

**Additional file 3**. Primers and PCR conditions of the *P. falciparum* and *P. vivax* *18sRNA* and anti-malarial drug resistance genes

| **PCR** | **Genes** | **Primer sequences (5' - 3')** | **Size**  **(bp)** | **Temp**  **(°C)** | **Time**  **(min)** | **Temp**  **(°C)** | **Time**  **(min)** | **Temp**  **(°C)** | **Time**  **(min)** | **No. of**  **cycles** | **Reference** |
| --- | --- | --- | --- | --- | --- | --- | --- | --- | --- | --- | --- |
| Primary | *18sRNA* | rPLU5: CCTGTTGTTGCCTTAAACTTC  rPLU6: TTAAAATTGTTGCAGTTAAAACG | 1200 | 94 | 1 | 58 | 2 | 72 | 5 | 30 | (Snounou *et al.,* 1993) |
| Nested | *pf18S* | rFAL-F: TTAAACTGGTTTGGGAAAACCAAATATATT  rFAL-R: ACACAATGAACTCAATCATGACTACCCGTC | 205 | 94 | 1 | 58 | 2 | 72 | 5 | 30 |  |
|  | *pv18S* | rVIV1: CGCTTCTAGCTTAATCCACATAACTGATAC  rVIV2: ACTTCCAAGCCGAAGCAAAGAAAGTCCTTA | 120 | 94 | 1 | 58 | 2 | 72 | 5 | 30 |  |
| Single | *pfcrt* | For: GGCTCACGTTTAGGTGGA  Rev: TGAATTTCCCTTTTTATTTCCAAA | 264 | 94 | 1 | 54 | 1 | 72 | 1.5 | 40 | (Vathsala *et al*., 2004) |
| Primary  Nested | *pfdhfr* | F1: TTTATGATGGAACAAGTCTGC  R1: CTAGTATATACATCGCTAACA  F2: TGATGGAACAAGTCTGCGACGTT  R2: CTGGAAAAAATACATCACATTCATATG | 594 | 94  94 | 1  1 | 52  52 | 2  2 | 72  72 | 1  1 | 40  40 | (Pearce *et al*., 2003) |
| Primary | *pfdhps* | F1: GATTCTTTTTCAGATGGAGG R1: TTCCTCATGTAATTCATCTGA |  | 94 | 0.5 | 47 | 0.5 | 72 | 1 | 35 | (Pearce *et al*., 2003) |
| Nested |  | F2: AACCTAAACGTGCTGTTCAA  R2: AATTGTGTGATTTGTCCACAA | 711 | 94 | 0.5 | 47 | 0.5 | 72 | 1 | 35 |  |
| Single^#^ | *pfmdr1* | For: ATGGGTAAAGAGCAGAAAGA  Rev: AACGCAAGTAATACATAAAGTCA | 603 | 94 | 1 | 55 | 1 | 72 | 1 | 38 | (Shrivastava *et al*., 2014) |
| Single^*^ | *pfmdr1* | For: AGAAGATTATTTCTGTAATTTGATAGAAAAAGC  Rev: ATGATTCGATAAATTCATCTATAGCAGCAA | 887 | 94 | 1 | 55 | 1 | 72 | 1 | 38 | (Isozumi *et* *al*., 2010) |
| Primary | *pfk13* | F1: GGGAATCTGGTGGTAACAGC  R1: CGGAGTGACCAAATCTGGGA | 2097 | 94 | 0.5 | 55 | 1 | 72 | 1 | 40 | (Ariey *et al*., 2014) |
| Nested |  | F2: GCCTTGTTGAAAGAAGCAGA  R2: GCCAAGCTGCCATTCATTTG | 849 | 94 | 0.5 | 55 | 1 | 72 | 1 | 40 |  |
| Single | *pvcrt-o* | For: TCCTTGCCGCTGATTCTACG  Rev: GGTAACGTTCATCGGGGGTT | 1194 | 95 | 0.5 | 60 | 1 | 72 | 1 | 35 | (Golassa *et al*., 2015) |
| Single | *pvdhfr* | For: ATGGAGGACCTTTCAGATGTATTTGACATT  Rev: TCACACGGGTAGGCGCCGTTGATCCTCGTG | 608 | 95 | 1 | 64 | 1.5 | 72 | 1.5 | 40 | (Sastu *et al*., 2016) |
| Primary | *pvdhps* | For: ATTCCAGAGTATAAGCACAGCACATTTGAG  Rev: CTAAGGTTGATGTATCCTTGTGAGCACATC |  | 94 | 0.5 | 58.8 | 0.7 | 72 | 1 | 34 | (Zakeri *et al*., 2010) |
| Nested |  | For: GATGGCGGTTTATTTGTCGAT  Rev: GCCTCCCCGCTCATCAGTCT | 767 | 94 | 0.5 | 56 | 0.7 | 72 | 1 | 35 | (Mint Deida *et al*., 2018) |
| Single | *pvmdr1* | For: CGCCATTATAGCCCTGAGCA  Rev: TGGTCTGGACAAGTATCTAAAA | 1057 | 95 | 1 | 58 | 1.5 | 72 | 1.5 | 40 | (Golassa *et al*., 2015; Cubides *et al*., 2018s) |
| Primary | *pvk12* | For: ATCCAACAGCATTTCCAACT  Rev: CAATTAAAACGGAATGTCCA |  | 95 | 0.5 | 54.4 | 1 | 72 | 1.5 | 24 | (Popovici *et al*., 2015) & Self-designed |
| Nested_1  Nested_2 |  | For: GAAAGGGAATCTGGGGCCAA  Rev: AAAACGGAATGTCCAAATCG  For: ACCACGTGACGAGGGATAAG  Rev: TTTCGAATATGGCTCCCCCG | 991  1015 | 95  95 | 0.5  0.5 | 58.8  52.6 | 1  1 | 72  72 | 1.5  1.5 | 34  34 | (Popovici *et al*., 2015) & Self-designed |

PCR: Polymerase chain reaction, *crt*: Chloroquine resistant transporter gene, *dhfr*: Dihydrofolate reductase gene, *dhps*: Dihydropteroate synthase gene, *mdr1*: Multidrug resistance protein 1 gene, *k13*: Kelch13 gene

^#^This set of primers was used to cover 86Y and 184F mutations in the *pfmdr1* gene

^*^This set of primers was used to cover 1034C, 1042D and 1246Y mutations in the *pfmdr1* gene

**References used**:

Ariey, F., Witkowski, B., Amaratunga, C., Beghain, J., Langlois, A.-C., Khim, N., Kim, S., & Duru, V. (2014). A molecular marker of artemisinin-resistant *Plasmodium falciparum* malaria. *Nature*, *505*, 50–55. https://doi.org/10.1038/nature12876.A

Isozumi, R., Uemura, H., Dao, L. D., Van Hanh, T., Giang, N. D., Vien, H. V., Phuc, B. Q., Van Tuan, N., & Nakazawa, S. (2010). Longitudinal survey of *Plasmodium falciparum* infection in Vietnam: Characteristics of antimalarial resistance and their associated factors. *Journal of Clinical Microbiology*, *48*(1), 70–77. https://doi.org/10.1128/JCM.01449-09

Pearce, R. J., Drakeley, C., Chandramohan, D., Mosha, F., & Roper, C. (2003). Molecular determination of point mutation haplotypes in the dihydrofolate reductase and dihydropteroate synthase of *Plasmodium falciparum* in three districts of northern Tanzania. *Antimicrobial agents and Chemotherapy*, 47(4), 1347–1354. https://doi.org/10.1128/AAC.47.4.1347-1354.2003

Shrivastava, S. K., Gupta, R. K., Mahanta, J., & Dubey, M. L. (2014). Correlation of molecular markers, *Pfmdr1*-N86Y and *Pfcrt*-K76T, with in Vitro chloroquine resistant *Plasmodium falciparum*, isolated in the malaria endemic states of Assam and Arunachal Pradesh, Northeast India. *PLoS ONE*, *9*(8):e0103848. https://doi.org/10.1371/journal.pone.0103848

Snounou Georges, Viriyakbosol Suganya, Zhu Xin Ping, Jarra William, Pinheiro Lucilia, Virgilio E. do Rosario, T. S. and B. K. N. (1993). High sensitivity of detection of human malaria parasites by the use of nested polymerase chain reaction. *Molecular and Biochemical Parasitology*, *61*, 315–320.

Vathsala, P. G., Pramanik, A., Dhanasekaran, S., Devi, C. U., Pillai, C. R., Subbarao, S. K., Ghosh, S. K., Tiwari, S. N., Sathyanarayan, T. S., Deshpande, P. R., Mishra, G. C., Ranjit, M. R., Dash, A. P., Rangarajan, P. N., & Padmanaban, G. (2004). Widespread occurrence of the *Plasmodium falciparum* chloroquine resistance transporter (*Pfcrt*) gene haplotype SVMNT in *P. falciparum* malaria in India. *The American Journal of Tropical Medicine and Hygiene*, 70(3), 256–259.

Cubides, J. R., Camargo-Ayala, P. A., Niño, C. H., Garzón-Ospina, D., Ortega-Ortegón, A., Ospina-Cantillo, E., Orduz-Durán, M. F., Patarroyo, M. E., & Patarroyo, M. A. (2018). Simultaneous detection of *Plasmodium vivax dhfr*, *dhps*, *mdr1* and *crt-o* resistance-associated mutations in the Colombian Amazonian region. *Malaria Journal*, 17:130. https://doi.org/10.1186/S12936-018-2286-5/TABLES/3

Golassa, L., Erko, B., Baliraine, F. N., Aseffa, A., & Swedberg, G. (2015). Polymorphisms in chloroquine resistance-associated genes in *Plasmodium vivax* in Ethiopia. *Malaria Journal*, 2015 14:1, 14: 164. https://doi.org/10.1186/S12936-015-0625-3

Mint Deida, J., Ould Khalef, Y., Mint Semane, E., Ould Ahmedou Salem, M. S., Bogreau, H., Basco, L., Ould Mohamed Salem Boukhary, A., & Tahar, R. (2018). Assessment of drug resistance associated genetic diversity in Mauritanian isolates of *Plasmodium vivax* reveals limited polymorphism. *Malaria Journal*, 17:416. https://doi.org/10.1186/S12936-018-2548-2

Popovici, J., Kao, S., Eal, L., Bin, S., Kim, S., & Ménard, D. (2015). Reduced polymorphism in the kelch propeller domain in *Plasmodium vivax* isolates from Cambodia. *Antimicrobial Agents and Chemotherapy*, 59(1), 730–733.

Sastu, U. R., Abdullah, N. R., Norahmad, N. A., Saat, M. N. F., Muniandy, P. K., Jelip, J., Tikuson, M., Yusof, N., & Sidek, H. M. (2016). Mutations of *pvdhfr* and *pvdhps* genes in vivax endemic-malaria areas in Kota Marudu and Kalabakan, Sabah. *Malaria Journal*, 15:63. https://doi.org/10.1186/S12936-016-1109-9

Zakeri, S., Afsharpad, M., Ghasemi, F., Raeisi, A., Safi, N., Butt, W., Atta, H., & Djadid, N. D. (2010). Molecular surveillance of *Plasmodium vivax dhfr* and *dhps* mutations in isolates from Afghanistan. *Malaria Journal*, 9:75. https://doi.org/10.1186/1475-2875-9-75
