# Supplementary material for: Nationwide spatiotemporal drug resistance genetic profiling from over three decades in Indian Plasmodium falciparum and Plasmodium vivax isolates
Source: Malar J. 2023 Aug 15;22:236. doi: 10.1186/s12936-023-04651-x (PMC10428610; doi:10.1186/s12936-023-04651-x)
Supplement: Supplementary file 4 — Additional file 4. Flow diagram showing number of samples analysed in the study for P. falciparum and P. vivax drug resistance genes. [file 12936_2023_4651_MOESM4_ESM.docx]

**Additional file 4**. Flow diagram showing number of samples analysed in the study for *P. falciparum* and *P. vivax* drug resistance genes


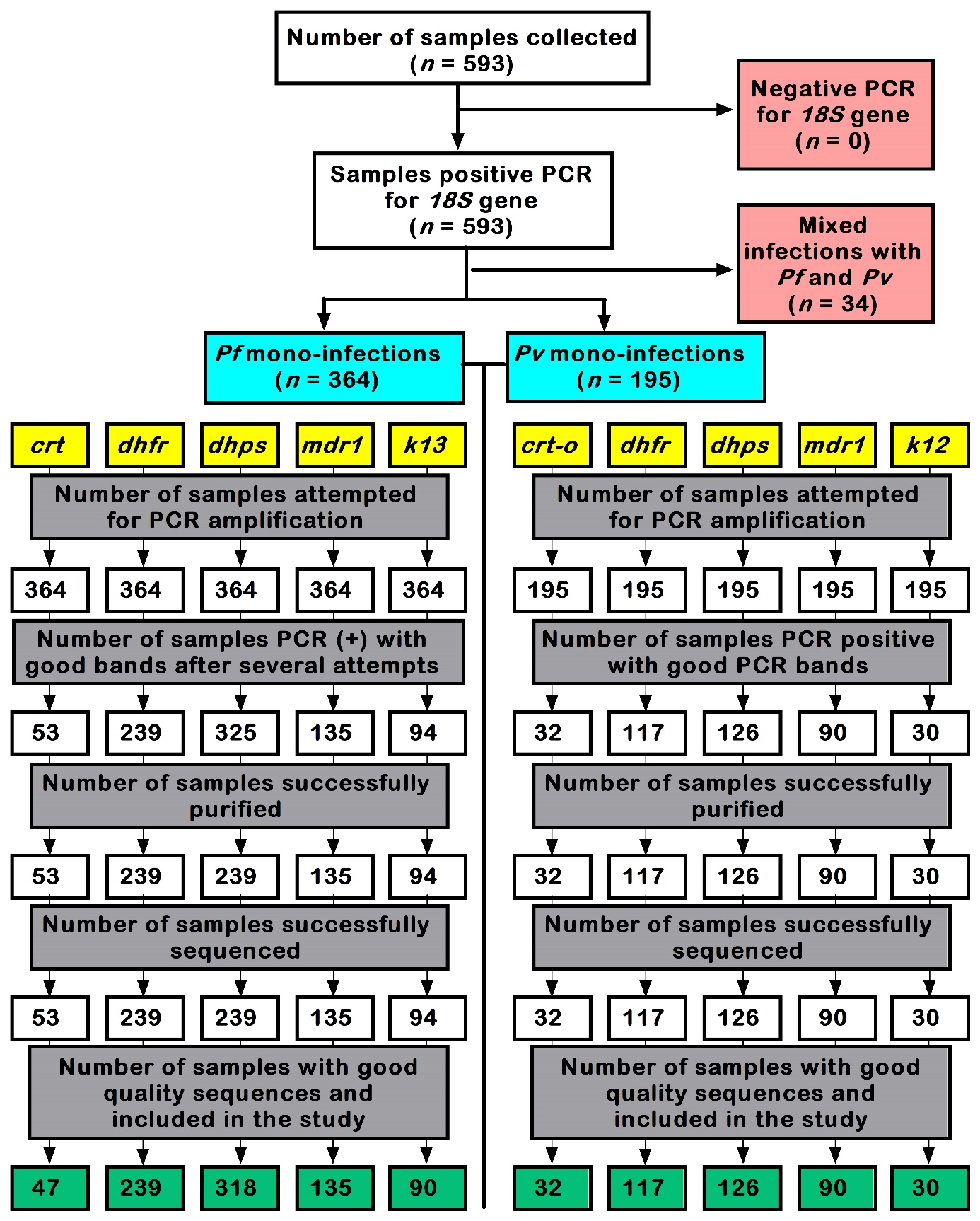


PCR: Polymerase chain reaction, *Pf*: *P. falciparum*, *Pv*: *P. vivax, crt*: Chloroquine resistant transporter gene, *crt-o*: Chloroquine resistant transporter orthologue gene, *dhfr*: Dihydrofolate reductase gene, *dhps*: Dihydropteroate synthase gene, *mdr1*: Multidrug resistance protein 1 gene, *k12*: Kelch12 gene, *k13*: Kelch13 gene
