# Supplementary material for: Nationwide spatiotemporal drug resistance genetic profiling from over three decades in Indian Plasmodium falciparum and Plasmodium vivax isolates
Source: Malar J. 2023 Aug 15;22:236. doi: 10.1186/s12936-023-04651-x (PMC10428610; doi:10.1186/s12936-023-04651-x)
Supplement: Supplementary file 5 — Additional file 5. Electrophoresis gels of different P. falciparum and P. vivax drug resistance gene amplicons. [file 12936_2023_4651_MOESM5_ESM.docx]

**Additional file 5**. Electrophoresis gels of different *P. falciparum* and *P. vivax* drug resistance gene amplicons


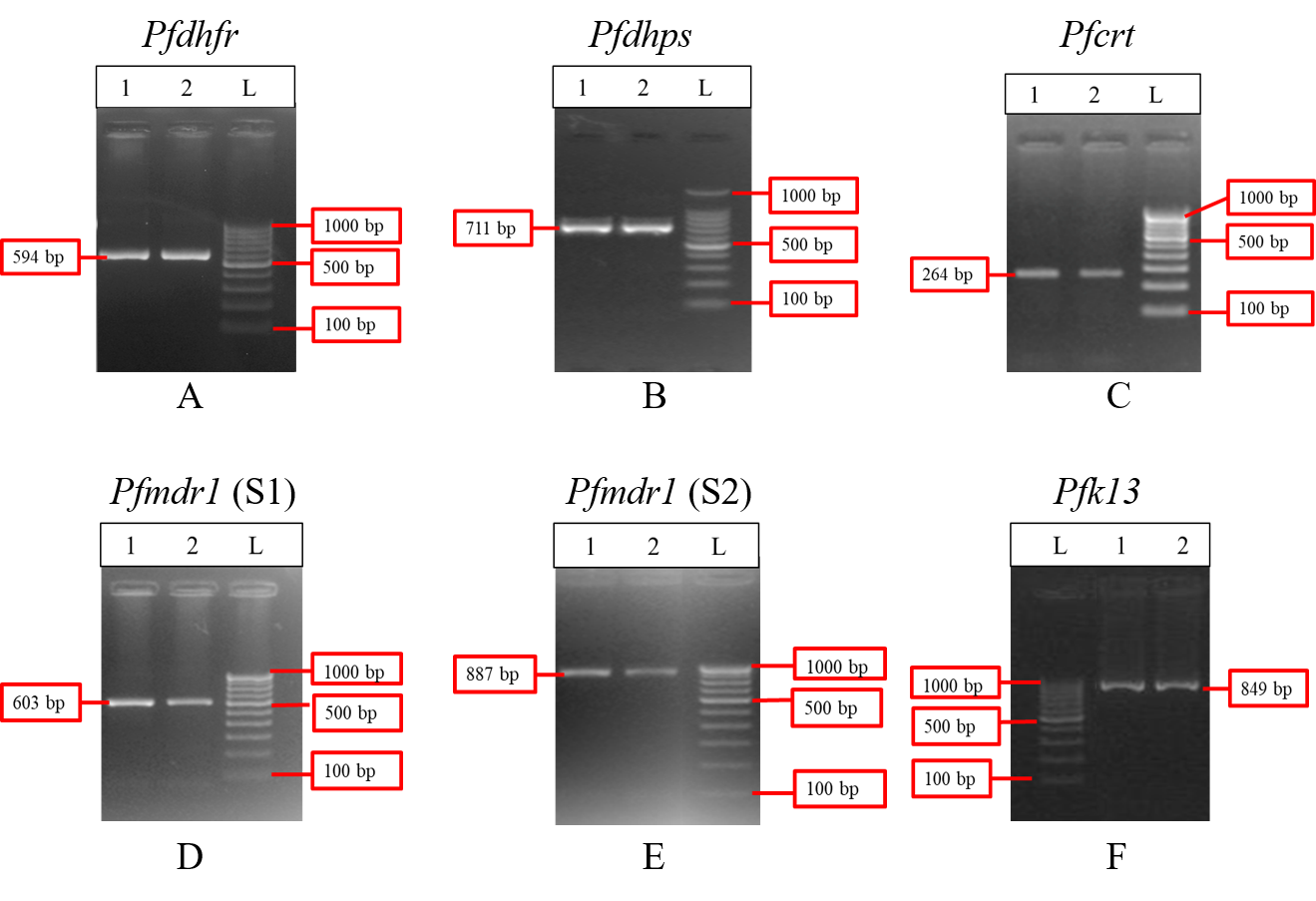


**Drug resistance genes for *Pf* gel image legend:**

The gel image displays the PCR amplification of different drug resistance gene in *Plasmodium falciparum*. Fig A to F shows PCR amplification of *pfdhfr, pfdhps, pfcrt, pfmdr1* (set-1 & 2) and *pfk13* gene respectively. There were two sets of primers for *pfmdr1* All Lane 1 shows the PCR product of an infected isolate and Lane 2 shows PCR product of positive control 3D7. Lane L is 100 bp ladder for reference.


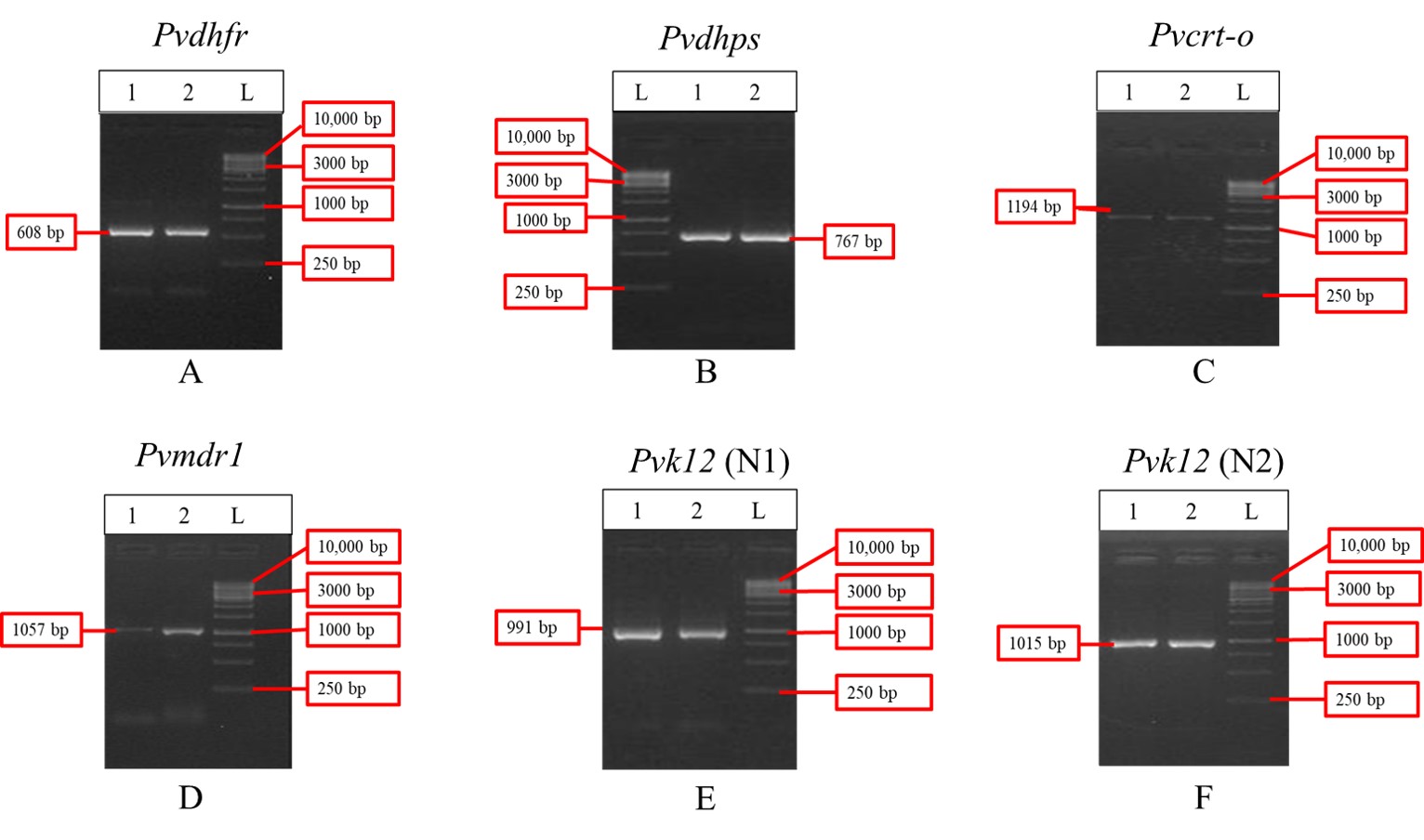


**Drug resistance genes for *Pv* gel image legend:**

The gel image displays the PCR amplification of different putative drug resistance markers in *Plasmodium vivax*. Fig A to F shows PCR amplification of *pvdhfr, pvdhps, pvcrt-o, pvmdr1* and *pvk12* (Nested set-1 & 2) gene respectively. There were two nested sets of primers for *pfk12.* All Lane 1 shows the PCR product of an infected isolate and Lane 2 shows PCR product of positive control 3D7. Lane L is 1 Kb ladder for reference.
