# Supplementary material for: Nationwide spatiotemporal drug resistance genetic profiling from over three decades in Indian Plasmodium falciparum and Plasmodium vivax isolates
Source: Malar J. 2023 Aug 15;22:236. doi: 10.1186/s12936-023-04651-x (PMC10428610; doi:10.1186/s12936-023-04651-x)
Supplement: Supplementary file 6 — Additional file 6. Resistance haplotypes by combining pfdhfr (codons 16, 50, 51, 59, 108, 164) and pfdhps (codons 431, 436, 437, 540, 581, 613). [file 12936_2023_4651_MOESM6_ESM.docx]

**Additional file 6**. Resistance haplotypes by combining *pfdhfr* (codons 16, 50, 51, 59, 108, 164) and *pfdhps* (codons 431, 436, 437, 540, 581, 613)

|  |  | **Years 1990 - 1999** | | |  | **Years 2000 - 2009** | | | |  | **Years 2010 - 2019** | | | |
| --- | --- | --- | --- | --- | --- | --- | --- | --- | --- | --- | --- | --- | --- | --- |
| **Haplotypes** | ***n*** | **DL-1994** | **MP-1996** | **OR-1995** |  | **DL-2000** | **OR-2001** | **DL-2008/10** | **GA-2008** |  | **CG-2011** | **MH-2012** | **JH-2013** | **HR-2015/16** |
| **Single (*n* = 13)** |  |  |  |  |  |  |  |  |  |  |  |  |  |  |
| acIcsi-isakaa | 4 | - | 2 (15.4%) | 1 (5.9%) |  | - | - | - | - |  | - | - | - | 1 (1.9%) |
| acncNi-isakaa | 4 | - | - | - |  | 2 (11.8%) | - | - | - |  | - | 2 (22.2%) | - | - |
| acnRsi-isakaa | 2 | 1 (8.3%) | - | 1 (5.9%) |  | - | - | - | - |  | - | - | - | - |
| acncsi-iAakaa | 1 | - | - | 1 (5.9%) |  | - | - | - | - |  | - | - | - | - |
| acncsi-isGkaa | 1 | - | - | - |  | - | - | - | 1 (25%) |  | - | - | - | - |
| acncsL-isakaa | 1 | - | - | - |  | - | - | - | - |  | - | - | - | 1 (1.9%) |
| **Double (*n* = 20)** |  |  |  |  |  |  |  |  |  |  |  |  |  |  |
| acncsi-iAaEaa | 3 | - | - | - |  | - | 1 (5.3%) | - | - |  | - | - | 2 (9.1%) | - |
| acnRsi-iAakaa | 3 | - | - | 1 (5.9%) |  | - | - | - | - |  | - | - | - | 2 (3.8%) |
| acnRNi-isakaa | 2 | - | 1 (7.7%) | - |  | - | - | 1 (2.7%) | - |  | - | - | - | - |
| acIcNi-isakaa | 1 | - | - | - |  | - | - | 1 (2.7%) | - |  | - | - | - | - |
| acIcsi-iAakaa | 1 | - | - | - |  | - | - | 1 (2.7%) | - |  | - | - | - | - |
| acIcsi-isGkaa | 1 | - | - | - |  | - | - | - | - |  | - | - | - | 1 (1.9%) |
| acIRsi-isakaa | 1 | - | - | - |  | - | - | 1 (2.7%) | - |  | - | - | - | - |
| acncNi-iAakaa | 1 | - | - | - |  | - | - | - | - |  | - | - | - | 1 (1.9%) |
| acncNi-isakGa | 1 | - | 1 (7.7%) | - |  | - | - | - | - |  | - | - | - | - |
| acncsi-iAakGa | 1 | - | - | - |  | - | - | 1 (2.7%) | - |  | - | - | - | - |
| acncsi-isGEaa | 1 | - | - | - |  | - | - | 1 (2.7%) | - |  | - | - | - | - |
| acncsi-isGkGa | 1 | - | - | - |  | - | - | 1 (2.7%) | - |  | - | - | - | - |
| acncsL-isakGa | 1 | - | - | - |  | - | - | - | - |  | - | - | - | 1 (1.9%) |
| acnRsi-isGkaa | 1 | - | - | - |  | - | - | - | - |  | - | - | - | 1 (1.9%) |
| acnRsL-isakaa | 1 | - | - | - |  | - | 1 (5.3%) | - | - |  | - | - | - | - |
| **Triple (*n* = 30)** |  |  |  |  |  |  |  |  |  |  |  |  |  |  |
| acncNi-iAaEaa | 9 | - | - | 1 (5.9%) |  | - | - | - | - |  | - | - | 8 (36.4%) | - |
| acnRNL-isakaa | 4 | - | 1 (7.7%) | - |  | 1 (5.9%) | - | 2 (5.4%) | - |  | - | - | - | - |
| acIcNi-isaEaa | 3 | 1 (8.3%) | - | - |  | - | - | - | - |  | - | - | - | 2 (3.8%) |
| acnRNi-isGkaa | 3 | - | - | - |  | - | - | - | 3 (75%) |  | - | - | - | - |
| acncsi-isGEaS | 2 | - | - | - |  | - | - | 2 (5.4%) | - |  | - | - | - | - |
| acnRNi-iAakaa | 2 | - | - | 1 (5.9%) |  | - | - | - | - |  | - | - | - | 1 (1.9%) |
| acnRsi-iAaEaa | 2 | - | - | - |  | 1 (5.9%) | - | - | - |  | - | - | - | 1 (1.9%) |
| acIcNi-isGkaa | 1 | - | 1 (7.7%) | - |  | - | - | - | - |  | - | - | - | - |
| acncNi-isGEaa | 1 | - | - | - |  | - | - | 1 (2.7%) | - |  | - | - | - | - |
| acncsi-iAaEGa | 1 | - | - | - |  | - | - | - | - |  | - | - | - | 1 (1.9%) |
| acnRsi-isGkGa | 1 | - | - | - |  | - | 1 (5.3%) | - | - |  | - | - | - | - |
| acnRsL-isGkaa | 1 | - | - | - |  | - | - | - | - |  | - | - | - | 1 (1.9%) |
| **Quadruple (*n* = 43)** |  |  |  |  |  |  |  |  |  |  |  |  |  |  |
| acnRNi-iAaEaa | 27 | - | - | 1 (5.9%) |  | 1 (5.9%) | - | 5 (13.5%) | - |  | 14 (53.8%) | - | 5 (22.7%) | 1 (1.9%) |
| acIcNi-iAaEaa | 2 | - | - | - |  | - | 1 (5.3%) | - | - |  | - | - | - | 1 (1.9%) |
| acIRNi-isaEaa | 2 | - | - | - |  | - | - | - | - |  | 1 (3.8%) | - | - | 1 (1.9%) |
| acIRsi-iAaEaa | 2 | - | - | - |  | 1 (5.9%) | - | 1 (2.7%) | - |  | - | - | - | - |
| acIcNi-isGkGa | 1 | - | - | - |  | 1 (5.9%) | - | - | - |  | - | - | - | - |
| acIcsL-isaEaS | 1 | - | - | - |  | - | - | - | - |  | - | - | - | 1 (1.9%) |
| acIcsL-isGEaa | 1 | - | - | - |  | - | - | - | - |  | - | - | - | 1 (1.9%) |
| acncNi-iAGEaa | 1 | - | - | - |  | - | - | - | - |  | - | 1 (11.1%) | - | - |
| acncNL-iAaEaa | 1 | - | - | - |  | - | - | - | - |  | - | - | 1 (4.5%) | - |
| acnRNi-isGkGa | 1 | - | - | - |  | - | - | - | - |  | - | - | - | 1 (1.9%) |
| acnRsL-iAGkaa | 1 | - | - | - |  | - | - | 1 (2.7%) | - |  | - | - | - | - |
| acnRsL-isaEGa | 1 | - | - | - |  | 1 (5.9%) | - | - | - |  | - | - | - | - |
| acnRsL-isGEaS | 1 | - | - | - |  | - | 1 (5.3%) | - | - |  | - | - | - | - |
| acnRsL-isGkGa | 1 | - | - | - |  | - | - | - | - |  | - | - | - | 1 (1.9%) |
| **Quintuple (*n* = 14)** |  |  |  |  |  |  |  |  |  |  |  |  |  |  |
| acnRNL-iAaEaa | 6 | - | - | - |  | - | 1 (5.3%) | 2 (5.4%) | - |  | 1 (3.8%) | - | 1 (4.5%) | 1 (1.9%) |
| acnRNi-iAaEaS | 4 | - | - | - |  | 1 (5.9%) | - | 2 (5.4%) | - |  | - | - | 1 (4.5%) | - |
| acIRNi-isGEaa | 1 | - | - | - |  | - | - | 1 (2.7%) | - |  | - | - | - | - |
| acnRNi-iAaEGa | 1 | - | - | - |  | - | - | - | - |  | 1 (3.8%) | - | - | - |
| acIRsL-isGkGa | 1 | - | - | - |  | - | - | - | - |  | - | - | - | 1 (1.9%) |
| acnRNL-isGEaa | 1 | - | - | 1 (5.9%) |  | - | - | - | - |  | - | - | - | - |
| **Sextuple (*n* = 4)** |  |  |  |  |  |  |  |  |  |  |  |  |  |  |
| acnRNL-iAaEaS | 2 | - | - | - |  | 1 (5.9%) | 1 (5.3%) | - | - |  | - | - | - | - |
| acIcNL-iAaEaS | 1 | - | - | - |  | - | - | - | - |  | - | - | - | 1 (1.9%) |
| acIRNi-iAaEaS | 1 | - | - | - |  | - | 1 (5.3%) | - | - |  | - | - | - | - |
| **Wild type (*n* = 105)** |  |  |  |  |  |  |  |  |  |  |  |  |  |  |
| acncsi-isakaa | 105 | 10 (83.3%) | 7 (53.8%) | 9 (52.9%) |  | 7 (41.2%) | 11 (57.9%) | 13 (35.1%) | 0 (0%) |  | 9 (34.6%) | 6 (66.7%) | 4 (18.2%) | 29 (54.7%) |
| **Total** |  | **12 (100%)** | **13 (100%)** | **17 (100%)** |  | **17 (100%)** | **19 (100%)** | **37 (100%)** | **4 (100%)** |  | **26 (100%)** | **9 (100%)** | **22 (100%)** | **53 (100%)** |

*Pf*: *P. falciparum*, *dhfr*: Dihydrofolate reductase gene, *dhps*: Dihydropteroate synthase gene

Wild alleles are in lower case and mutated alleles are in upper case

The international codes of Indian areas were used as abbreviations. CG: Chhattisgarh, DL: Delhi, GA: Goa, HR: Haryana, JA: Jharkhand, MH: Maharashtra, MP: Madhya Pradesh, OR: Orissa/Odisha
